# A covalent and cleavable antibody-DNA conjugation strategy for sensitive protein detection via immuno-PCR

**DOI:** 10.1038/srep22675

**Published:** 2016-03-07

**Authors:** Jessie A. G. L. van Buggenum, Jan P. Gerlach, Selma Eising, Lise Schoonen, Roderick A. P. M. van Eijl, Sabine E. J. Tanis, Mark Hogeweg, Nina C. Hubner, Jan C. van Hest, Kimberly M. Bonger, Klaas W. Mulder

**Affiliations:** 1 Radboud University, Faculty of Science, Radboud Institute for Molecular Life Sciences, Department of Molecular Developmental Biology, Nijmegen, the Netherlands; 2 Radboud University, Faculty of Science, Radboud Institute for Molecular Life Sciences, Department of Biomolecular Chemistry, Nijmegen, the Netherlands; 3 Radboud University, Faculty of Science, Institute for Molecules and Materials, Department of Bio-organic Chemistry, Nijmegen, the Netherlands; 4 Radboud University, Faculty of Science, Radboud Institute for Molecular Life Sciences, Department of Molecular Biology, Nijmegen, the Netherlands

## Abstract

Immuno-PCR combines specific antibody-based protein detection with the sensitivity of PCR-based quantification through the use of antibody-DNA conjugates. The production of such conjugates depends on the availability of quick and efficient conjugation strategies for the two biomolecules. Here, we present an approach to produce cleavable antibody-DNA conjugates, employing the fast kinetics of the inverse electron-demand Diels-Alder reaction between tetrazine and *trans*-cyclooctene (TCO). Our strategy consists of three steps. First, antibodies are functionalized with chemically cleavable NHS-s-s-tetrazine. Subsequently, double-stranded DNA is functionalized with TCO by enzymatic addition of N_3_-dATP and coupling to *trans-*Cyclooctene-PEG_12_-Dibenzocyclooctyne (TCO-PEG_12_-DBCO). Finally, conjugates are quickly and efficiently obtained by mixing the functionalized antibodies and dsDNA at low molar ratios of 1:2. In addition, introduction of a chemically cleavable disulphide linker facilitates release and sensitive detection of the dsDNA after immuno-staining. We show specific and sensitive protein detection in immuno-PCR for human epidermal stem cell markers, ITGA6 and ITGB1, and the differentiation marker Transglutaminase 1 (TGM1). We anticipate that the production of chemically cleavable antibody-DNA conjugates will provide a solid basis for the development of multiplexed immuno-PCR experiments and immuno-sequencing methodologies.

Antibody-DNA conjugate based technologies are used in biomedical research and the food industry to detect and quantify specific proteins or molecules^1^. In these technologies, antibody-conjugated DNA can be detected via gel electrophoresis^2^,^3^, fluorescence hybridization^4^, sequencing^5^,^6^ or quantitative polymerase chain reaction (immuno-PCR)^7^ after antibody binding to the targeted epitopes. In order to develop and implement such technologies, it is essential to produce antibody-DNA conjugates with the following characteristics. First, the conjugation approach itself should be (cost-)efficient and applicable to all antibodies. Secondly, the produced conjugates have to maintain specificity for their targeted epitope. Finally, sensitive detection of the DNA should be facilitated by release of the DNA barcode after immuno-staining. The antibody and DNA conjugation strategies that are available include non-covalent strategies, such as coupling via biotin-streptavidin^3^ or covalent conjugation, using e.g. thiol-maleimide chemistry^2^. To find an antibody-DNA conjugation strategy that facilitates all of the previously mentioned characteristics, however, is a major challenge. Yet such a strategy is critical to attain efficient and cleavable conjugation of any antibody.

Antibody-DNA conjugation depends on the production of antibodies and DNA with functional chemical groups. Antibody functionalization can be achieved via enzymatic reactions^8^, chemical tagging^9^,^10^,^11^ or incorporation of non-natural amino acids^7^. These approaches can be laborious and are not necessarily applicable to a wide variety of commercially available antibodies. In contrast, *N*-Hydroxysuccinimide ester (NHS) chemistry makes use of available primary amine groups present in all antibodies, and is therefore widely applied to generate antibody-fluorophore conjugates for microscopy and fluorescence activated cell sorting (FACS). DNA functionalization can be achieved by either incorporation of modified dNTPs during chemical synthesis of an oligonucleotide, enzymatic reactions such as PCR, or end labelling. Notably, PCR is a cost-efficient and renewable source of dsDNA for conjugation.

We aimed to develop an easy and efficient protocol for conjugation of antibodies to double stranded DNA (dsDNA). In the past decade, a wide variety of bioorthogonal reactions have been developed that allow conjugation of biomolecules^12^,^13^,^14^, including the Staudinger ligation^15^, Cu(I)-catalyzed azide-alkyne (CuAAC)^16^,^17^, strain-promoted azide-alkyne cycloaddditon (SPAAC)^18^ and inverse electron-demand Diels-Alder (iEDDA) reaction^19^,^20^. From these reactions, the iEDDA reaction between tetrazine and *trans-*cyclooctene (TCO) displays one of the fastest reaction constants, estimated at ~2,000–20,000 M^−1^s^−1^ 20, making it a very suitable candidate for the conjugation of antibodies and dsDNA.

Making use of 1) the robustness of NHS-chemistry for antibody functionalization with tetrazine, 2) the cost-efficient production of TCO-dsDNA and 3) the quick reaction kinetics of tetrazine with TCO, we developed an efficient procedure to conjugate specific dsDNA sequences to a set of different antibodies. Furthermore, we included a disulphide-containing cleavable linker between NHS and tetrazine to allow highly efficient release of dsDNA using DTT and highly sensitive DNA detection in qPCR after immuno-staining (Fig. 1). We obtained between 50- and 100-fold signal over background in immuno-PCR with conjugates against human epidermal (skin) stem cell markers integrin α6 (ITGA6), integrin β1 (ITGB1) or differentiation marker Transglutaminase 1 (TGM1). Antibody and cell dilution series, as well as siRNA silencing experiments showed sensitive and specific protein detection in immuno-PCR using these conjugates. The approach described in this article can in principle be used to conjugate dsDNA to any antibody, and is thus broadly applicable to many different fields of research or industry where specific and sensitive protein detection via immuno-PCR is of interest.

## Results

### Functionalization of antibodies with tetrazine using NHS-chemistry

We aimed to develop an antibody-dsDNA conjugation approach applicable to a broad spectrum of (commercially) available antibodies. Ideally, such an approach should not require production of modified recombinant antibodies, laborious enzymatic modifications or other specialized methods that can only be applied to a selection of specific antibodies. Due to the universal presence of primary amines on antibody molecules we chose the widely used NHS chemistry as an antibody functionalization approach. In addition, we wanted to combine this functionalization strategy with bioorthogonal chemistry, allowing selective conjugation of the antibody with other biomolecules, in our case dsDNA. We first tested the applicability of the SPAAC and iEDDA reactions for antibody conjugation to polyethylene glycol (PEG_5000_) by coupling different functional groups to these two molecules. We functionalized a mouse monoclonal antibody (against protein Transglutaminase 1, TGM1) with bicyclononyne (BCN), norbornene (Norb), TCO or tetrazine using NHS chemistry, followed by a conjugation reaction with N_3_-, tetrazine- or TCO-functionalized PEG_5000_ for 1 hour or overnight. We found that BCN-, TCO- or tetrazine-functionalized antibody required only 1 hour incubation to conjugate tetrazine-PEG_5000_ or TCO-PEG_5000_ respectively (Supplementary Fig. S1), although the exact time for conjugation with antibodies may be different. In contrast, BCN- or norbornene-functionalized antibodies required overnight incubation with tetrazine- PEG_5000_ or N_3_-PEG_5000_ respectively. Due to very fast kinetics^20^ and the higher stability of TCO compared to BCN^21^, we chose to continue with the iEDDA reaction between TCO and tetrazine for the remainder of the work.

**Figure 2:**
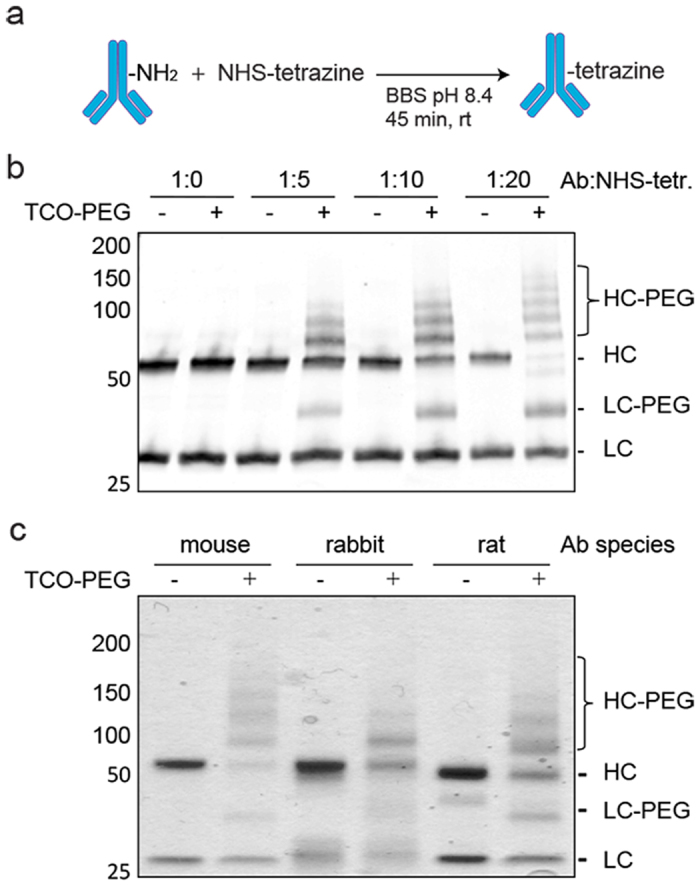
Optimization of antibody functionalization with NHS-PEG_4_-tetrazine (Figs 3,1). (**a**) Reaction conditions of antibody functionalization with tetrazine via NHS chemistry. (**b**) Western blot with ratio series of mouse (anti-TGM1) antibody: NHS-tetrazine (Figs 3,1), and conjugation with TCO-PEG_5000_. (**c**) Coommassie staining of SDS-PAGE with mouse (anti-TGM1), rabbit (IgG) or rat (Ago2) antibody functionalized using a 5-fold excess of NHS-tetrazine (Figs 3,1) and conjugation with TCO- PEG_5000_.

**Figure 3:**
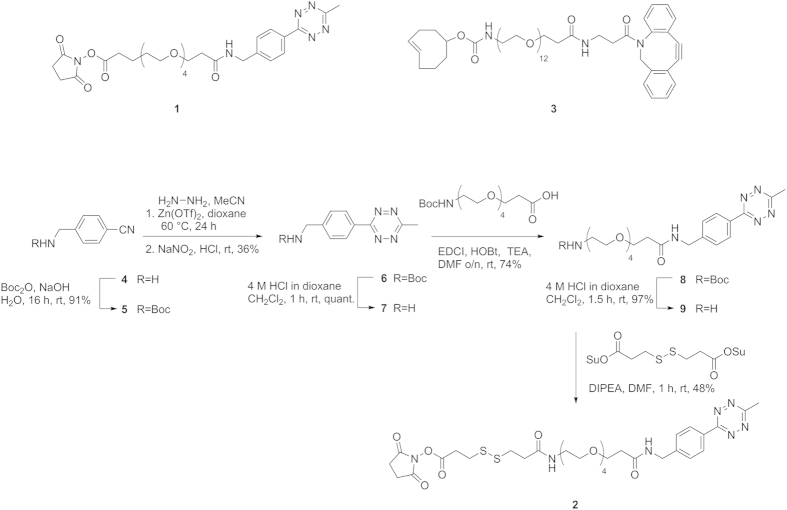
Structure of NHS-PEG_4_-tetrazine (1), and synthesis route towards NHS-s-s-PEG_4_-tetrazine (2), and structure of DBCO-PEG_12_-TCO (3). The details of the synthesis route to **2** are described in the Supplementary experimental section.

We proceeded to optimize the antibody functionalization reaction for our antibodies (Fig. 2a) with NHS-tetrazine **1** (Fig. 3). The NHS-chemistry used for the antibody functionalization reaction is dependent on 1) the available lysines of the antibody, 2) the pH of the buffer and 3) on the ratio of the antibody and the NHS-ester. Functionalization reactions were performed in borate buffered saline (BBS) at pH 8.4 for 45 minutes at room temperature (rt). The functionalization efficiency of antibody to NHS-tetrazine was compared in a molar ratio series of antibody:NHS-tetrazine, and assessed by the conjugation to TCO-PEG_5000_ followed by Western blot analysis. A higher molar ratio of antibody: NHS-tetrazine results in an increased number PEG_5000_ on the heavy chain of the TGM1 antibodies (Fig. 2b). We found that only a minor proportion of the light chain of the antibody is functionalized using different ratios. To test whether the functionalization approach is applicable to antibodies derived from different animal hosts, we functionalized mouse (TGM1) and rat monoclonal as well as rabbit polyclonal antibodies with NHS-tetrazine (at a molar ratio of 1:5), followed by conjugation with TCO-PEG_5000_. Western blot analysis revealed that all three types of antibodies were functionalized and conjugated (Fig. 2c).

After optimizing functionalization conditions, we explored the use of NHS-s-s-PEG_4_-tetrazine **2** (Fig. 3) in our functionalization strategy. **2** contains a disulphide bond between the NHS and tetrazine groups, which allows the controlled release of conjugated DNA from antibodies under reducing conditions. We first synthesized tetrazine **6**, which was prepared from **4** according to modified literature procedures^22^,^23^. Coupling of **6** to a Boc-protected PEG-linker resulted in **8** which, after Boc removal, was coupled to a bifunctional NHS-dithiopropionate to afford the target NHS-s-s-PEG_4_-tetrazine **2** (Supporting info, experimental section). We observed similar functionalization efficiencies for both non-cleavable NHS-PEG_4_-tetrazine **1** and cleavable NHS-s-s-PEG_4_-tetrazine **2**, as determined by Western blotting of non-reducing SDS-PAGE (Supplementary Fig. S2).

**Figure 4:**
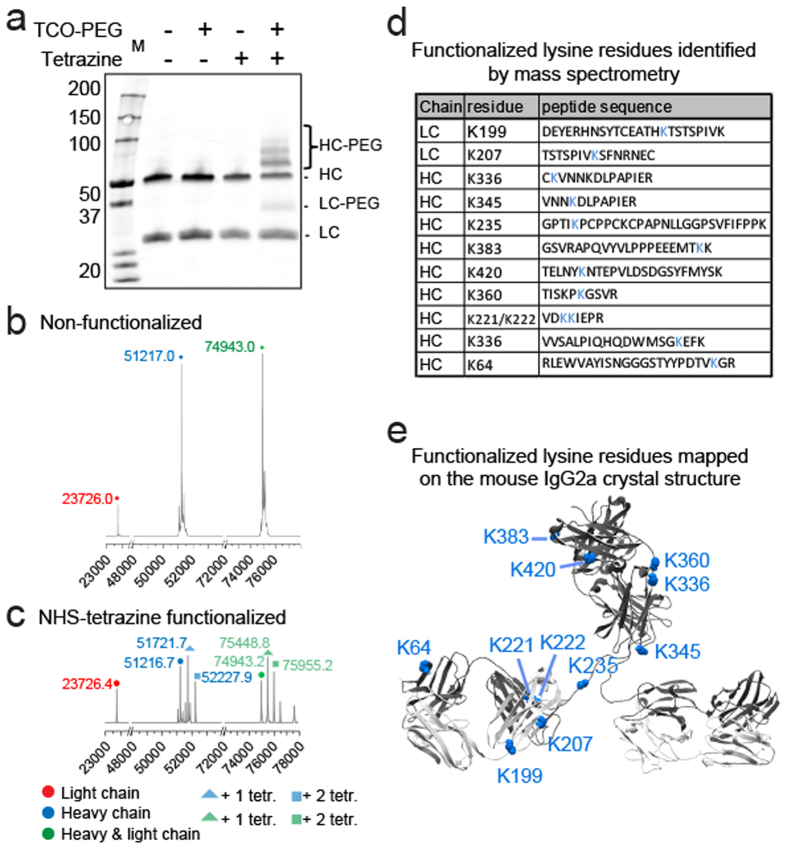
Characterization of functionalized antibody using electrospray ionization time-of-flight (ESI-TOF) mass spectrometry and LC-MS/MS. (**a**) Western blot of functionalized antibody (anti-TGM1) and conjugation with TCO-PEG_5000_. (**b,c**) Non-functionalized and functionalized mouse antibody (anti-TGM1) analysed by ESI-TOF. (**d**) Overview functionalized lysine residues and peptides of functionalized anti-TGM1 antibody analysed by LC-MS/MS. (**e**) Mapping of functionalized residues on mouse IgG2a. Structure and amino acid numbering based on PDB file 1IGT. HC = heavy chain, LC = light chain.

To determine the number of tetrazine-groups after functionalization of a batch of antibodies, we performed Western blot analysis in parallel to electrospray ionization time-of-flight (ESI-TOF) mass spectrometry of reduced functionalized antibodies (molar ratio 1:5, Fig. 4a). ESI-TOF mass spectrometry showed that each antibody heavy chain contained up to three functional groups (Fig. 4b,c and Supplementary Fig. S3). This is similar to the amount of PEG_5000_ groups conjugated to the heavy chain observed in the Western blot of the same sample conjugated to PEG_5000_ (Fig. 4a). These results show that the Western blot of PEG-conjugated antibodies can be used to determine the number of functional groups on the antibodies.

Given that the NHS-chemistry targets primary amines, there are numerous potential functionalization sites present in each antibody molecule. To characterize the potential positions of the modified amino acid residues, we performed the following experiment. First, we functionalized a mouse IgG2a monoclonal antibody with a ten-fold molar excess of the cleavable NHS-s-s-Tetrazine **2**. The functionalized antibody was denatured, digested with trypsin/lysC and reduced using DTT. This procedure leads to cleavage (reduction) of not only the disulphide bridges within the antibody, but also within the linker. Finally, the sample including reduced modified lysines was alkylated using iodoacetamide. These steps lead to a 145.02 Da ‘fingerprint’ on the functionalized lysines and a missed-cleavage of these peptides, allowing identification of modified sites using high resolution mass spectrometry (LC-MS/MS) (Fig. 4d). We mapped the identified modification sites on the crystal structure of mouse IgG2a and observed a total of nine modified lysines on the heavy-chain and two on the non-variable (non-epitope binding) part of the light-chain (Fig. 4d,e). As expected, all these modifications are positioned on the solvent-exposed surface of the antibody. Although the exact positions of the modified residues will be different for each antibody, our results suggest that any antibody that contains surface exposed lysines can be functionalized with a limited number of tetrazine groups via NHS-chemistry.

### Development of an easy dsDNA functionalization approach

**Figure 5:**
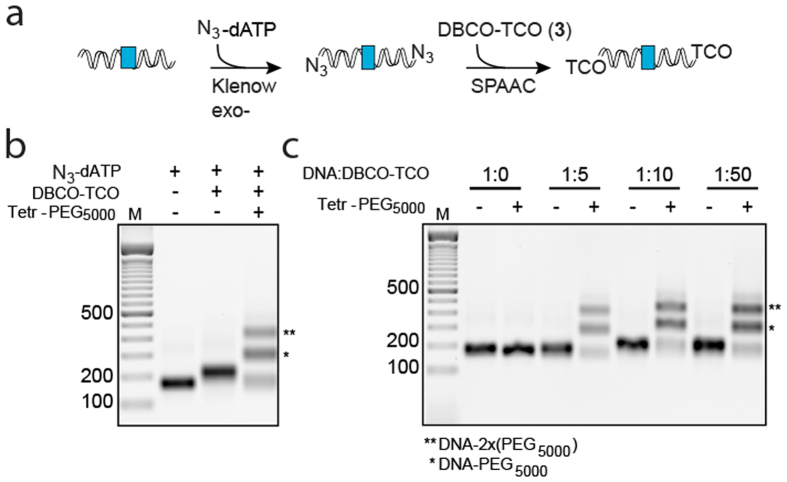
Production of functionalized dsDNA with TCO using Klenow exo- fragment and SPAAC. (**a**) Enzymatic addition of N_3_-dATP to dsDNA via Klenow exo- fragment and SPAAC for functionalization with TCO. (**b**) Agarose gel with SYBR Green stained dsDNA of 128 bp before and after conjugation. (**c**) Agarose gel with SYBR Green stained N_3_-dsDNA after ratio series with DBCO- PEG_12_-TCO **2** and conjugation to tetrazine-PEG_5000_. The first lane contains a DNA ladder.

To introduce functional groups on dsDNA that are compatible with iEDDA, we developed a combined enzymatic and chemical functionalization approach. After production of a blunt-ended PCR product, the 3′-ends of the dsDNA PCR product were extended with a single N_3_-dATP (azide-dATP) using *E. coli* DNA polymerase I Klenow fragment lacking 3′ → 5′ exonuclease activity (Fig. 5a). This polymerase makes use of blunt-ended dsDNA and adds specifically one dATP to the 3′-ends of the dsDNA. N_3_-labelled dsDNA was subsequently conjugated to bifunctional DBCO-PEG_12_-TCO **3** (Fig. 3) through a SPAAC reaction, leading to a shift in migration in an agarose gel. Conjugation to tetrazine-PEG_5000_ and analysis on agarose gel showed a near complete functionalization of the dsDNA with one or two TCO moieties (Fig. 5b).

To optimize the functionalization efficiency we performed a molar ratio series of N_3_-dsDNA to DBCO- PEG_12_-TCO **3** and monitored conjugation via gel electrophoresis. We found that high functionalization efficiency is achieved with mild (five to ten fold) excess of **3** (Fig. 5c), facilitating easy and efficient removal of non-conjugated **3** using a gel filtration column. Thus, dsDNA produced via a regular PCR reaction can be efficiently functionalized by combining enzymatic incorporation of N_3_-dATP and conjugation of TCO via SPAAC chemistry. In contrast to modified ssDNA oligo’s, our dsDNA production and functionalization strategy can be used on any blunt-end dsDNA PCR product, and allows the production of functionalized DNA in large quantities. By using unique DNA sequences per antibody, one could develop multiplexed immuno-PCR.

### Conjugation of antibody and dsDNA using the iEDDA

**Figure 6:**
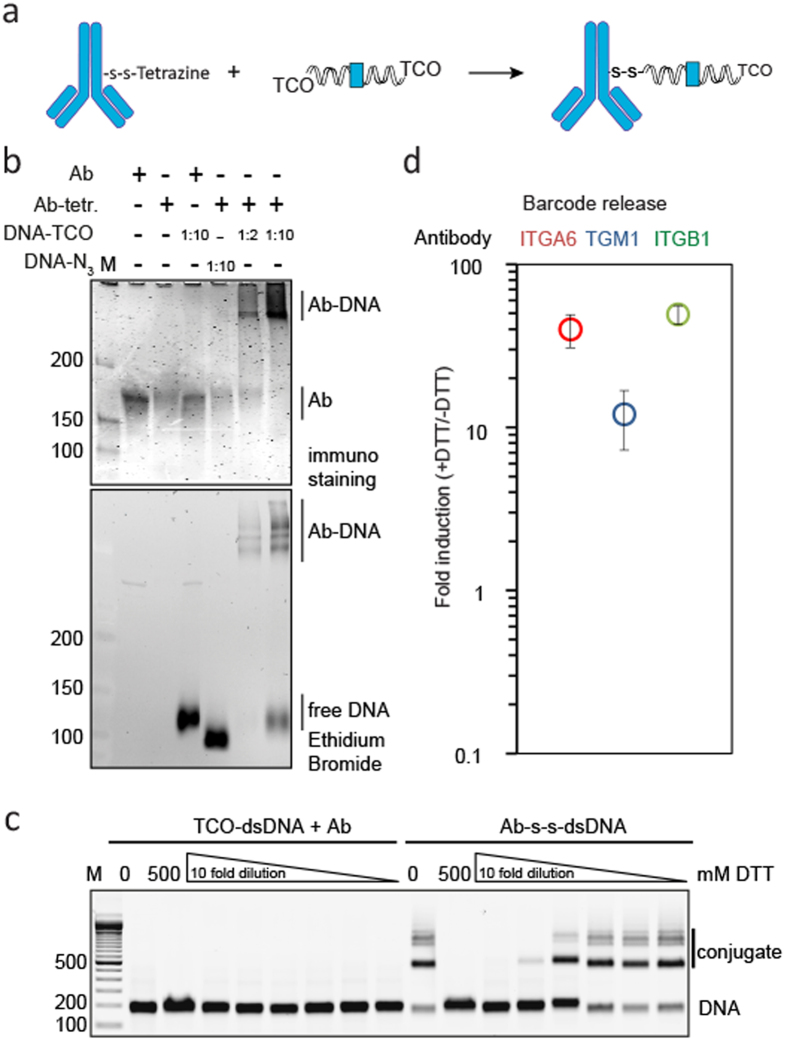
Production of cleavable antibody-dsDNA conjugates using the inverse electron-demand Diels-Alder reaction. (**a**) Schematic overview of antibody and dsDNA conjugation. Conjugates are blocked with free tetrazine. (**b**) Immuno staining (In gel western) and ethidium bromide stained 4–15% polyacrylamide gel, showing anti-TGM1 antibody or dsDNA respectively. (**c**) Agarose gel of SYBR Green stained dsDNA and conjugates, after concentration series of DTT treatment. (**d**) qPCR analysis after DTT treatment of immuno-stained keratinocytes with ITGA6 (n = 3), TGM1 (n = 6) or ITGB1 (n = 3) conjugates.

After functionalization of antibody and dsDNA with tetrazine and TCO respectively, we aimed to determine conditions that facilitate efficient conjugation of the two biomolecules (Fig. 6a). First, we determined the time needed for efficient conjugation, using NHS-PEG_4_-tetrazine **1**. Gel electrophoresis shows that the reaction is saturated within 30 minutes, which underlines the fast reaction kinetics of TCO with tetrazine (Supplementary Fig. S4). For the conjugation of antibodies with DNA we used a reaction time of one or two hours for further conjugation reactions, followed by quenching of the remaining TCO groups with free tetrazine. Because the functionalized dsDNA has one or potentially two functional groups per molecule, quenching of the TCO groups is desirable to prevent sequential conjugation of antibodies and dsDNA over time.

Next, we determined the conjugation efficiency at both the DNA and antibody level. The conjugates were visualized by running the samples on a 4–15% polyacrylamide gradient gel, followed by in-gel antibody-staining with fluorescently labelled antibodies, and subsequent DNA-staining with ethidium bromide. We observed conjugation at molar ratios of 1:2 and 1:10 antibody to DNA. These conjugates were seen at the same position in the polyacrylamide gradient gel via immuno-staining and via ethidium bromide staining (Fig. 6b). Taken together, the characterization of the conjugates directed us to use a molar ratio of antibody to dsDNA of 1:2 for the production of the following conjugates.

To determine whether functionalized and conjugated antibodies maintain their specificity, antibodies against two skin stem cell markers, integrin α6 (ITGA6) and integrin β1 (ITGB1) and one differentiation marker Transglutaminase I (TGM1), were used for immuno-staining (in-cell Western). We observed loss of signal for unconjugated, NHS-PEG_4_-tetrazine functionalized and dsDNA-conjugated antibodies following siRNA silencing of the targeted epitopes (Supplementary Fig. S5), indicating that the antibodies maintain their specificity after functionalization and conjugation.

Next, we aimed to determine the optimal conditions for the release of the DNA, without interfering with downstream PCR analysis. A disulphide bridge containing linker between antibody and DNA allows DNA release upon the presence of DTT. An advantage of a s-s containing linker over photo-cleavable linker^2^ is that the cleavage only occurs in presence of DTT without the risk of light-dependent instability issues during handling of the conjugates. Moreover, the DTT reduces all disulphide bridges, including the ones of the antibodies. Another advantage is that there is no extra risk of light-induced DNA-damage when using DTT to release the DNA. The release efficiency could be dependent on DTT concentration and availability of the conjugates. To test which concentration of DTT is needed to release the DNA, antibody-dsDNA conjugates were prepared using NHS-s-s-PEG_4_-tetrazine **2**, and subsequently incubated with decreasing concentrations of the reducing agent DTT. Gel electrophoreses showed that at DTT concentrations exceeding 5 mM, most DNA is effectively released from the antibodies (Fig. 6c). Moreover, DTT concentrations up to 50 mM did not affect the efficiency of subsequent DNA amplification by qPCR (Supplementary Fig. S6). Based on these results, we chose to use 10 mM DTT for dsDNA release after immunostaining.

To confirm the detection of barcodes after immunostaining and DTT treatment we produced antibody-dsDNA conjugates ITGA6, ITGB1 and TGM1 (Supplementary Fig. S7), and used these conjugates in an immuno-staining on fixed human epidermal stem cells. dsDNA was released using 10 mM DTT for 2 hours at rt and measured with quantitative PCR. Compared to control samples without DTT, we observed 39.8 fold (p = 0.0018) higher signal in samples stained with ITGA6 and 49.3 fold (p = 0.002) ITGB1 conjugates, and 12.0 fold (p = 0.0002) higher signal for samples stained with TGM1 conjugates. The cells that were used for this experiment where undifferentiated skin stem cells, which could explain the lower signal of differentiation marker TGM1. Together, these results provide a workflow for creating cleavable antibody-DNA conjugates that can be directly detected in qPCR after standard immuno-staining and DTT mediated release of the dsDNA.

### Sensitive detection of human skin stem cell and differentiation markers via immuno-PCR using DTT cleavable antibody-DNA conjugates

**Figure 7:**
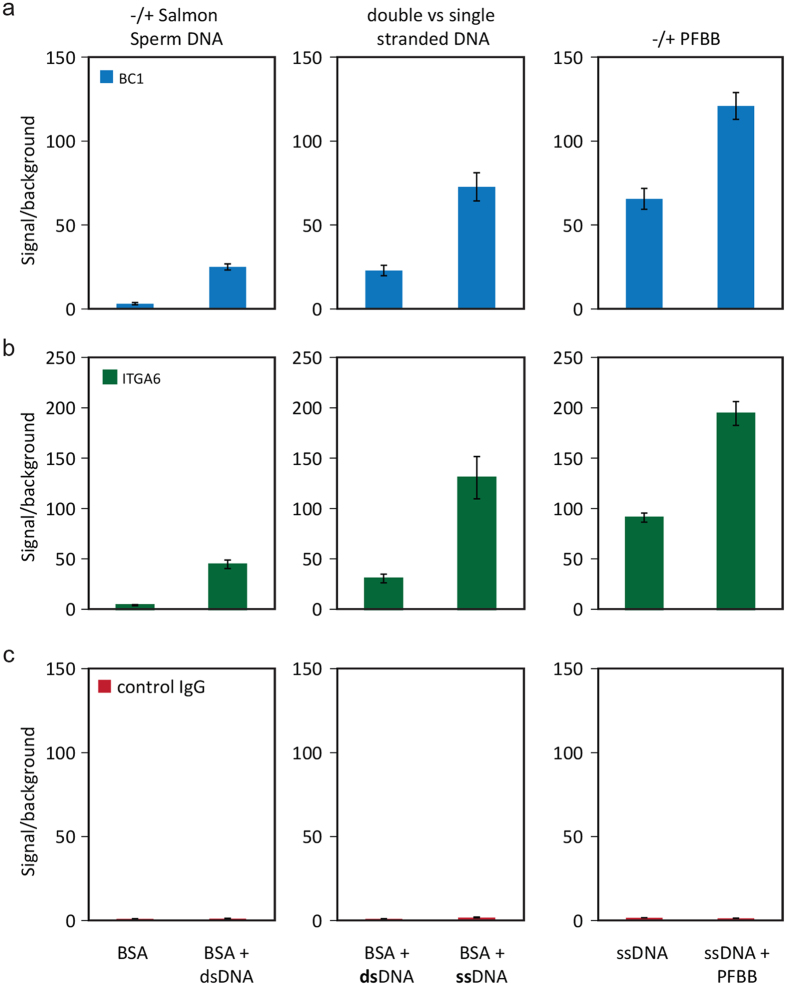
Optimization of the immune-PCR protocol by using several blocking conditions. The signal (2^−Ct^) is normalized over unconjugated dsDNA (background) signal. Anti-TGM1 (**a**) ITGA6 (**b**) or unspecific IgG (**c**) were used in the immuno-PCR (N = 4). DNA = Salmon sperm DNA, ds = double stranded, ss = single stranded, PFBB = protein free blocking buffer.

After developing a protocol for antibody-dsDNA conjugation and release, we optimized the immunostaining procedure using three conjugates for TGM1, ITGA6, or control IgG (Supplementary Fig. S7b). In the immuno-PCR, unspecific antibody binding or unspecific DNA binding could contribute to high background signal, and would result in lower signal over background levels. To reduce background signal from our conjugates in immuno-PCR, several blocking conditions were tested in immunostaining. We tested the influence of double or single stranded salmon sperm DNA and the effect of a protein free blocking reagent on the signal over background (Fig. 7a–c). The background in this experiment was defined as the mean signal coming from cell populations stained with unconjugated dsDNA. First, addition of double stranded salmon sperm DNA to our ‘standard’ blocking solution for ICW (1% bovine serum albumin in PBS) increased signal over background to >25 for the two specific antibodies TGM1 and ITGA6 (Fig. 7a,b, left column). Second, a further increase to >75 signal over background was achieved when using single stranded, rather than double stranded, salmon sperm DNA (Fig. 7a,b, middle column). Finally, the highest signal over background (120 and 194 for the TGM1 and ITGA6 antibody conjugates, respectively) was obtained when combining single stranded salmon sperm DNA with protein free blocking buffer instead of 1% bovine serum (Fig. 7a,b, right column). In all conditions the control IgG-DNA conjugate showed low signal of <1.6 (Fig. 7c). This is two orders of magnitude lower than the specific antibodies, indicating little unspecific binding events of the conjugates.

The specificity of two conjugates TGM1 and ITGA6 was validated by performing an immuno-PCR on cell populations with or without siRNA silencing of the targets TGM1 and ITGA6 respectively. Compared to control cells, a significant decrease of the protein level was detected using our conjugates in immuno-PCR (Supplementary Fig. S8). The mRNA levels of TGM1 and ITGA6 in these cells were determined using quantitative reverse transcription PCR (RT-qPCR), confirming efficient silencing of the mRNA. Together, these results show that the conjugates specifically recognize their targets in immuno-PCR.

**Figure 8:**
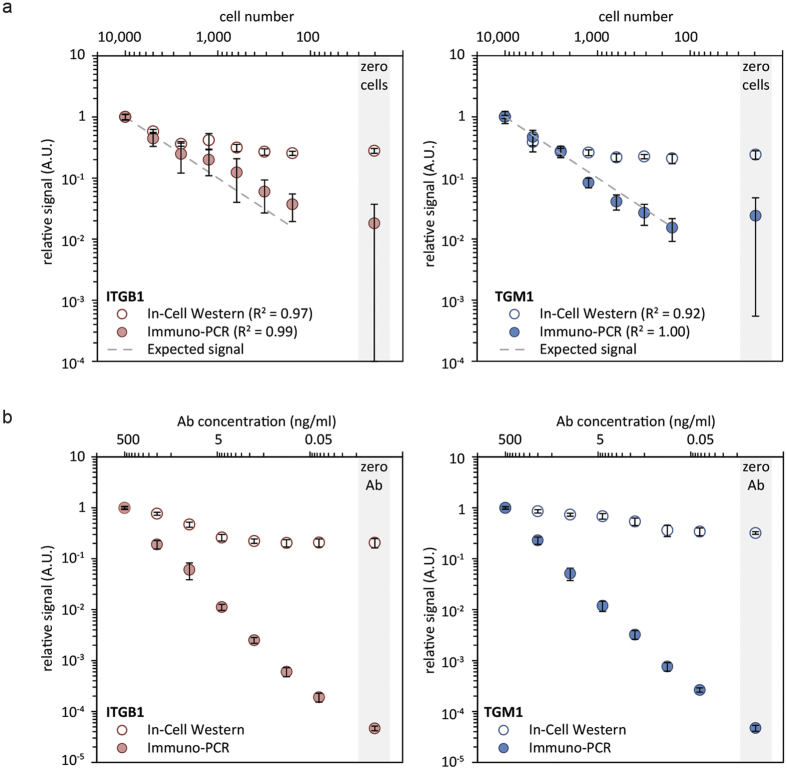
Comparing in-cell western and immuno-PCR approach to detect ITGB1 and TGM1. (**a**) Relative signal (to first dilution, A.U.: Arbitrary Unit) from cell-dilution series in ICW and immuno-PCR of ITGB1 (n = 6) and TGM1 (n = 5). (**b**) Relative signal (to first dilution, A.U.: Arbitrary Unit) from an antibody dilution series (log_10_ μg/ml) in ICW and immuno-PCR of ITGB1 and TGM1 (n = 6).

Finally we evaluated the sensitivity of two different conjugates in the immuno-PCR. First, we fixed epidermal stem cell populations containing different cell numbers and thus different amounts of epitopes. Then, we determined the protein levels of TGM1 or ITGB1 via immuno-PCR using antibody-DNA conjugates or via a standard in-cell western (ICW) using unconjugated antibodies (Fig. 8a). The relative limit of detection (LOD) in the ICW is 0.358 and 0.353 for ITGB1 and TGM1 respectively. The immuno-PCR approach, however, has a lower LOD of 0.095 and 0.094 for ITGB1 and TGM1 respectively. Moreover, the squared correlation coefficient to the 2-fold dilution factor is higher with the immuno-PCR approach (ITGB1: R^2^ = 0.99, TGM1: R^2^ = 1.00) than with the ICW (ITGB1: R^2^ = 0.97, TGM1: R^2^ = 0.92). Together, these results show that immuno-PCR is able to detect much lower signal than with ICW. Secondly, we performed a dilution series of the antibody-DNA conjugates (Fig. 8b) to determine how little of the antibodies is needed for detection above background. The background signal from cell-populations without antibody (‘no antibody’) is much lower with immuno-PCR than with ICW (ITGB1: ~4400 and TGM1: ~6800 times lower background). We observed in immuno-PCR a log-linear relationship between antibody concentration and signal over 3 orders of magnitude before approaching the background signal coming from cell-populations without antibody (Fig. 8b). This indicates that very low concentrations (total of 1.6 ng per 50 μl, in lowest dilution) of our conjugates are sufficient for detection of proteins through immuno-PCR. To test the usefulness of our conjugation and immuno-PCR method for other (intracellular) proteins, we have performed similar antibody-dilution experiments using a wide variety of conjugates against >40 (mostly intracellular) proteins (data not shown). The average squared correlation of these antibodies to the dilution factor is 0.988 with a standard deviation of 0.036, indicating our conjugation and immuno-PCR approach is applicable to many different antibodies. Together, these results show that our antibody-dsDNA can be used for sensitive immuno-PCR experiments and that a comparatively small amount of conjugates is needed in these experiments.

## Conclusion

We have developed a strategy for antibody and dsDNA conjugation and sensitive immuno-PCR experiments. The approach consists of an easy to apply antibody functionalization step and two-step dsDNA functionalization, followed by conjugation of the two molecules via tetrazine and TCO. By introducing a DTT cleavable linker, dsDNA can be released after immuno-staining for sensitive detection in qPCR. Distinct sequences of dsDNA can be conjugated to the antibodies, which would allow the development of multiplexed immuno-PCR experiments. The throughput of the strategy may be increased by performing reactions in parallel and in a miniaturized, or automated, fashion. We believe the described conjugation strategy for DTT-cleavable antibody-DNA conjugates is an important step towards easy implementation of high-throughput multiplexed immuno-staining analysis by quantitative PCR and potentially by high-throughput sequencing.

### Experimental Procedures

#### Antibodies

Rat IgG, referring to an antibody against Argonaute2, was obtained from Sigma (Clone11A9, Cat. No. SAB4200085). Purified Rabbit IgG was obtained from Bethyl laboratories (Cat. No. P120-101). Antibodies against ITGA6 (clone MP4F10) and ITGB1 (clone P5D2) were a kind gift from Simon Broad. GAPDH antibody was obtained from Abcam (clone 6C5).

The antibody that was used for functionalization experiments (in figures referred to as ‘anti-TGM1′ or TGM1) was produced from mouse hybridoma line BC.1 (recognizing Transglutaminase I); Hybridoma cells were cultured in RPMI medium 1640 + GlutaMAX^TM^-I (Gibco life technologies) supplemented with Penicillin/Streptavidin (P/S) and 10% fetal bovine serum (FBS, Lonza) for 4 days. Then cells were passaged every 3 days in this medium with 5%, 2,5% or 1% FBS. After 13 days cells were passaged and resuspended at 10^6^ cells/mL in PFHM-II medium + P/S. Culture medium containing the antibody was harvested after 9 days. Antibody was purified over ProtA/G column (GE Healthcare) at 4 °C. 50 K Amicon filter (Millipore) and 40 K Zeba^TM^ Spin Desalting columns (Thermo Scientific) were used for buffer exchange into PBS.

#### Functionalization of antibodies

For all antibodies, a buffer exchange to 50 mM borate buffered Saline pH 8.4 (150 mM NaCl) was performed using 40 K Zeba^TM^ Spin Desalting columns (Thermo Scientific). Antibodies (1.5–2 μg/ul) were incubated for 45 minutes with NHS-PEG_4_-tetrazine **1** (Jena Bioscience) or NHS-s-s-tetrazine **2** (Fig. 3, production of **2** see supplementary ‘Experimental section”) in the indicated molar ratios at rt. Surplus **1** or **2** was removed using 40 K Zeba^TM^ Spin Desalting columns. Functionalized antibodies were stored in 50 mM borate buffered Saline pH 8.4 (150 mM NaCl) or PBS at 4 °C or −20 °C.

#### Mass spectrometry ESI-TOF

Protein mass characterization was performed by electrospray ionization time-of-flight (ESI-TOF) on a JEOL AccuTOF CS. Deconvoluted mass spectra were obtained using MagTran 1.03 b2. Protein samples were desalted and concentrated to 10–100 μM by spin filtration (amicon 10 K filter from millipore) with MQ.

#### Mass spectrometry LC-MS/MS

To determine the localization of tetrazine modifications, 1 μg of functionalized antibody in 1 ul was diluted in 15 μL of 8 M Urea in 100 mM Tris, pH 8. Disulphide bonds were reduced by adding 2 ul 10 mM dithiothreitol and subsequently alkylated by adding 2 ul 50 mM iodoacetamide, for 15 minutes in the dark. Subsequently, the antibody was digested using 0.5 ul of Trypsin/Lys-C mix (0.04 μg/ul, Promega) overnight at rt. The digestion was stopped by acidifying with trifluoroacetic acid and the peptides were purified on StageTips^24^. Thirty percent of the peptides were loaded onto a pulled fused silica column (New Objectives) packed in house with 1.8 μm Reprosil-Pur C18-AQ (Dr. Maisch, 9852). Using the Easy-nLC 1000 (Thermo Fisher Scientific), peptides were separated in a 60 min. gradient and directly injected into a QExactive mass spectrometer (Thermo Fisher Scientific). The mass spectrometer was operated in TOP10 data dependent acquisition. Full MS were recorded at a resolution of 70,000 at *m/z* = 400 and a scan range of 300–1,650 *m/z*. MS/MS spectra were recorded at a resolution of 17,500. Raw mass spectrometry data was analyzed using the MaxQuant software package, version 1.5.0.0, with standard settings if not further specified^25^. The following variable modifications were allowed: Oxidation of methionines, acetylation of protein *N*-termini, and carbamylation of cysteines. Furthermore, a modification of lysines and protein *N*-termini corresponding to the reduced and alkylated linker (Δm = 145.01975) was allowed. This modification was only allowed for peptide internal lysines due to the missed trypsin cleavage that is caused by the linker modification. Three missed cleavages were allowed and the maximum peptide mass was set to 8000 Dalton. Data was searched against the mouse Uniprot database (downloaded 13.06.2014) using the integrated Andromeda search engine. The search was performed with a mass tolerance of 4.5 ppm mass accuracy for the precursor ion and 20 ppm for fragment ions. Peptides, modified peptides and proteins were accepted at an FDR of 0.01.

#### Production of TCO-PEG_12_-dsDNA

Template and primers (for sequences see Table S1) were ordered from Biolegio and were used for standard PCR reaction to produce dsDNA barcode-1, 2 or 3 using Pfu proof-reading DNA polymerase that produces blunt-ended dsDNA. After purification using a PCR purification kit (Qiagen), a Klenow exo- (New England Biolabs) enzymatic reaction was used to add N_3_-dATP (Jena Bioscience) to the 3′-end of the barcodes. For this, up to 8 μg dsDNA per reaction was incubated for 1 hour (h) at 37 °C. Following a second purification using PCR purification kit (Qiagen), SPAAC was used for functionalization of N_3_-dsDNA with DBCO-PEG_12_-TCO (Jena Bioscience) using a molar ratio of 1:20. After overnight reaction at rt, surplus DBCO-PEG_12_-TCO was removed using a Zeba^TM^ Spin desalting column (Thermo Scientific; 40 KDa molecular-weight cut-off).

#### Conjugation conditions reverse electron-demand Diels-Alder chemistry

To determine functionalization efficiency of TCO-dsDNA or tetrazine-antibodies, tetrazine-PEG_5000_ or TCO-PEG_5000_ were conjugated to dsDNA or antibodies respectively (molar ratio 1:300, 1 h at rt). Conjugation of antibody and dsDNA was performed with a molar ratio of 1:2 in PBS for 1 h at rt (ITGA6, ITGB1 in Fig. 6c,d, Supplementary Figs S4 and S5) or with a molar ratio of 4:1 in BBS pH 8.4 for 2 h at rt (ITGA6, anti-TGM1, control IgG in Fig. 7 and anti-TGM1 in Fig. 6c and all conjugates in Fig. 8 and Supplementary Figs S7 and S8), unless stated otherwise. Conjugation reactions were quenched by addition of an excess of 3,6-diphenyl tetrazine. Conjugation reactions can be linearly scaled from 1 to 100 μg antibody with similar conjugation efficiencies.

#### Gel electrophoresis

dsDNA and dsDNA-PEG_5000_ were run with 10× SYBR Green I (Life technologies) on a 2% agarose gel (0.5 × TBE) and scanned on a Typhoon Trio+ machine (GE Healthcare).

#### Western blots

After 10 minutes incubation with 1x sample buffer (1% SDS, 40 mM TrisHCl pH 6.8, 5% glycerol, β-ME, BPB) at 95 °C, antibodies were separated on standard 4–15% gradient gel (Biorad) and blotted on a nitrocellulose or PVDF membrane using Bio-Rad Trans-Blot^®^ Turbo RTA Transfer Kit (mixed molecular weight program). Antibodies were detected with specific fluorescent goat-anti-mouse antibody (1:10.000, Licor).

#### In gel western

After 5 minutes incubation with 2 × non-reducing sample buffer (1% SDS, 40 mM TrisHCl pH 6.8, 5% glycerol) at 95 °C, antibody-DNA conjugates were run on a standard 4–15% gradient gel (Biorad) at constant 20 mA for 1.5 to 2 h. Then, the gel was fixed with 50% propanol, 5% acitic acid for 15 minutes. After 3 washes with MQ, the gel was incubated with fluorescent secondary antibody (anti-mouse IRDYe800, 1:2000) overnight at 4 °C. Following three 10 minutes washes with PBST, the gel was washed with PBS and scanned on odyssey CLx (LI-COR). Then DNA was stained using 20 minutes incubation with 1 μg/mL ethidium bromide in PBS (gel was fully submerged in solution). After two washes with PBS, the gel was imaged using Gel Doc XR+ (BioRAD).

#### Cell culture and siRNA transfection

Primary pooled human keratinocytes (foreskin strain Knp) were obtained from Lonza. Cells were expanded and cultured as described^26^. Before transfection, expanded keratinocytes were grown for several days in keratinocyte serum-free medium (KSFM) supplemented with 30 μg/mL bovine pituitary extract and 0.2 ng/mL EGF (Gibco) until 70% confluency. After collection, cells were resuspended in cell line buffer SF (Lonza) with 2 × 10^5^ cells per 18 μL. Then, 2 × 10^5^ cells in SF were mixed with 2 μL siRNA (20 μM) and transfected (program FF-113) using the Amaxa 96 well shuttle system (Lonza). After 10 minutes incubation, cells were resuspended in KSFM and seeded in 96 wells plate at 20.000 cells per well. Cells were grown for 48 h, washed with 150 ul PBS and fixed with 50 ul 4% formaldehyde in PBS for 10 minutes at rt.

#### Immunostaining (in-cell-western)

Procedure for Fig. S5: Fixed keratinocytes (as described under cell culture and siRNA transfection) were washed 3 times with 150 μl PBS and permeabilized with 0.1% triton for 10 minutes at rt. After blocking overnight with 10% bovine serum in PBS, cells were incubated with control or DNA-conjugated antibodies for 1 h at rt at 2 μg/ml (ITGA6, ITGB1) or ~ 0.3 μg/ml (TGM1). Cells were washed with PBS (3 × 10 minutes) followed by secondary antibody staining with Goat-anti-mouse 1:2000 and DRAQ5 1:4000 in blocking buffer. Analysis was performed with an Odyssey scanner. Measurements of the total intensity were normalized over DRAQ5 and the average and standard deviation were calculated and plotted as the relative intensity.

Procedure for Fig. 8: cells were washed 1× with 150 μl PBS and fixed with 4% formaldehyde for 15 minutes. After 3 washes with 150 μL TBS, cells were incubated for 30 minutes with blocking and permeabilization buffer (0.5 × protein free blocking buffer, 0.1% triton, 200 ng/ml single strand Salmon Sperm DNA). Fixed cells were incubated with 50 μl primary antibodies (TGM1 or ITGB1 antibody in 0.5 × protein free blocking buffer, 0.1% triton) at 0.1 μg/ml for the cell dilutions series or at the indicated concentration for 2 h at rt. The staining was followed by 3 × short washes, 1 × 15 minutes wash, 3 × short washes with PBS. Cells were then incubated with 50 μl Goat-anti-mouse 1:2000 and DRAQ5 1:4000 in blocking buffer. Analysis was performed with an Odyssey scanner.

#### Barcode release and immuno-PCR

Cells were cultured in a 96 wells plate (15.000 or 20.000/well) for 2–3 days, washed with PBS and fixed with 4% formaldehyde for 15 minutes and stored in PBS at 4 °C after 3 washes with 150 μL PBS. Then cells were incubated for 30 minutes with blocking and permeabilization buffer (0.1% triton, 0.5 × protein free blocking buffer (PFBB), 200 ng/ml single strand Salmon Sperm DNA). For optimization of blocking conditions the following buffers were tested: 0.1% triton with 2% BSA in PBS or 0.5 × PFBB in PBS, unboiled (double strand) salmon sperm DNA or boiled (single stranded) salmon sperm DNA (200 ng/ml).

Cells were incubated with 50 μL primary antibody conjugates at 0.5 μg/mL unless stated otherwise for 2 h at rt. Subsequently cells were washed three times with 150 μL blocking/permeabilization buffer for 15 minutes. After three times rinsing with PBS, barcodes were released using 50 μl of 10 mM DTT (in 150 mM borate buffered saline, 50 mM NaCl) for 2 h at rt. After thorough vortexing, 2 μL sample was used for quantitative PCR (20 μL/reaction, iQ^TM^ SYBR Green Supermix, CFX 96 machine). To avoid template contamination, it is important to work carefully by using filter tips and regularly rinsing the working area.

### Data analysis

qPCR data in Fig. 6: Each signal (Ct) was divided by the mean signal from immuno-stained cells treated without DTT. The average of 3 (ITGA6 and ITGB1) or 6 (TGM1) replicates is shown in the figure with the corresponding standard deviation. The p-value described is calculated by a t-test (2-tailed, equal variance)

qPCR data in Fig. 7: Each signal (Ct) was divided by the mean signal form cells that were stained with unconjugated dsDNA. The average of 4 replicates with the standard deviation is plotted in the figure.

qPCR data in Fig. 8: The average, standard deviation and coefficient of variation of 5 replicates was calculated. Per technique (ICW or IPCR) signals are plotted relative to the first and highest signal, the arbitrary unit (A.U.), in order to compare the signals from the two techniques.

## Additional Information

**How to cite this article**: van Buggenum, J. A. G. L. *et al.* A covalent and cleavable antibody-DNA conjugation strategy for sensitive protein detection via immuno-PCR. *Sci. Rep.* **6**, 22675; doi: 10.1038/srep22675 (2016).

## Supplementary Material

Supplementary Information

## Figures and Tables

**Figure 1:**
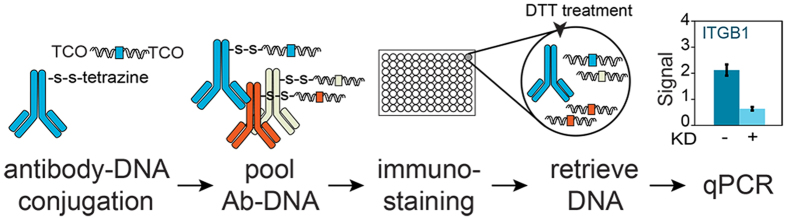
Overview of Immuno-PCR method using antibody-dsDNA conjugates. Last schematic graph shows signal (2^−Ct^) of two cell populations: without (−) and with (+) knockdown (KD) of the measured protein integrin β1 (ITGB1).
