# Supplementary material for: A covalent and cleavable antibody-DNA conjugation strategy for sensitive protein detection via immuno-PCR
Source: Sci Rep. 2016 Mar 7;6:22675. doi: 10.1038/srep22675 (PMC4780193; doi:10.1038/srep22675)
Supplement: Supplementary Information [file srep22675-s1.pdf]

Supporting information

A covalent and cleavable antibody-DNA conjugation strategy for sensitive protein detection via immuno-PCR.

Jessie A.G.L. van Buggenum[1], Jan P. Gerlach[1], Selma Eising[2], Lise Schoonen[3], Roderick A.P.M. van Eijl[1], Sabine E. Tanis[1], Mark Hogeweg[1], Nina C. Hubner[4], Jan C. van Hest[3], Kimberly M. Bonger[2] and Klaas W. Mulder[1,*]

[1]Radboud University, Faculty of Science, Radboud Institute for Molecular Life Sciences, Department of Molecular Developmental Biology, Nijmegen, the Netherlands. [2]Radboud University, Faculty of Science, Radboud Institute for Molecular Life Sciences, Department of Biomolecular Chemistry, Nijmegen, the Netherlands. [3]Radboud University, Faculty of Science, Institute for Molecules and Materials, Department of Bio-organic Chemistry Nijmegen, the Netherlands. [4]Radboud University, Faculty of Science, Radboud Institute for Molecular Life Sciences, Department of Molecular Biology, Nijmegen, the Netherlands.

[*]Corresponding author, k.mulder@science.ru.nl

Table of contents

Experimental section 3

1. General experimental 3
2. Synthesis 4
3. NMR spectra 6

Supporting Figures 12

References 21

Experimental section

1. General experimental

Unless stated otherwise, all chemicals were obtained from Sigma-Aldrich and used without further purification. $Zn(OTf)_2$ and anhydrous DMF were obtained from Acros Organics, $NaNO_2$ and NaOH were purchased from J. T. Baker. MeOD-d4 was obtained from Cambridge Isotope Laboratories, Inc., 1-ethyl-3-(3-dimethylaminopropyl)carbodiimide was purchased from Chem-Impex International, Inc. Solvents were purchased from J.T. Baker, Biosolve or Fisher Scientific and used as received. If no further details are given the reaction was performed under ambient atmosphere and temperature. Analytical thin layer chromatography (TLC) was performed on silica gel-coated plates (Merck, 60 F254) with the indicated solvent mixture, visualization was done using ultraviolet (UV) irradiation (λ = 254 nm) and/or staining with aqueous $KMnO_4$. Purification by column chromatography was carried out using silica gel 60 (Merck, 0.040-0.063 mm). ^{1}H NMR spectra were recorded on a Bruker DMX 300 (300 MHz) or an Advance III Bruker 500 (500 MHz) spectrometer in $CDCl_3$ or MeOD-d4. TMS (δH 0.00) or the NMR solvent residual peak of $CHCl_3$ (δH 7.26) or MeOH (δH 3.31) were used as the internal reference. Proton coupling constants (Hz) of the phenyl protons were determined by computer simulation using MestReNova. ^{13}C NMR spectra were recorded on a Bruker DMX 300 (75 MHz) or an Advance III Bruker 500 (125 MHz) spectrometer in $CDCl_3$ or MeOD-d4, using the central resonance of $CDCl_3$ (δC 77.2) or MeOD-d4 (δC 49.0) as the internal reference. Low-resolution mass spectra (LRMS) were recorded on Thermo LCQ Advantage Max (ESI). High-resolution mass spectra (HRMS) were recorded on a JEOL AccuTOF JMS-T100CS (Electrospray Ionization (ESI).

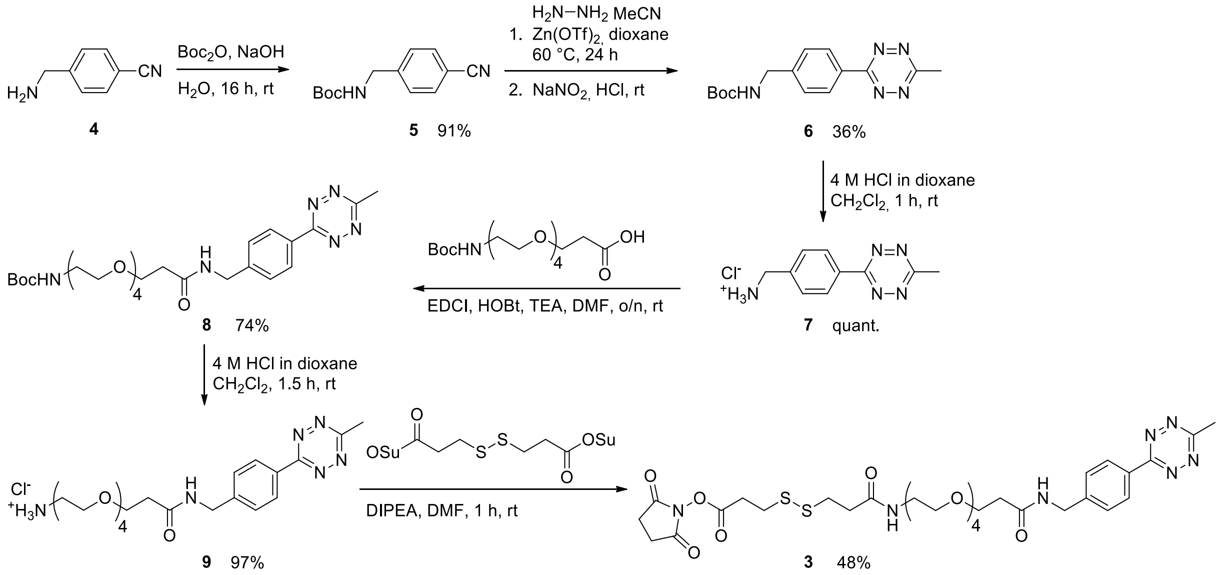

Supporting Scheme 1. Synthetic route towards dithiol-containing tetrazine **3**

2. Synthesis

***tert*-Butyl 4-cyanobenzylcarbamate (5).** The synthesis was performed according to a published procedure.[1]

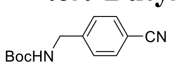

4-(Aminomethyl)benzonitrile hydrochloride **4** (4.10 g, 24.3 mmol, 1.0 equiv.) in H_2O (25 mL) was added to di-*tert*-butyl dicarbonate (6.2 mL, 27.0 mmol, 1.1 equiv.) and NaOH (2.93 g, 73.3 mmol, 3.0 equiv.) in H_2O (25 mL). This mixture was stirred for 16 h. The white solid was filtered off, washed twice with H_2O and lyophilized, yielding carbamate **5** (5.12 g, 22.0 mmol, 91%) as a white solid. ^{1}H NMR (300 MHz, $CDCl_3$) δ: 7.61 and 7.38 (AA'XX' pattern, J_{AX} = 8.0 Hz, $J_{AA'}$ = 1.8 Hz, $J_{XX'}$ = 1.7 Hz, $J_{AX'}$ = 0.4 Hz, 2H), 5.00 (br s, 1H), 4.36 (d, J = 6.1 Hz, 2H), 1.45 (s, 9H). ^{13}C NMR (75 MHz, $CDCl_3$) δ: 156.0, 144.8, 132.5, 127.9, 118.9, 111.2, 80.2, 44.3, 28.5. LRMS (ESI+) *m/z* calcd. for $C_{10}H_{11}N_5$ $[M+H]^+$ 233.1, found: 232.7

***tert*-Butyl 4-(6-methyl-1,2,4,5-tetrazin-3-yl)benzylcarbamate (6).** Cyanide **5** (1.00 g, 4.31

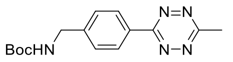

mmol, 1.0 equiv.), acetonitrile (2.25 mL, 43.1 mmol, 10.0 equiv.) and $Zn(OTf)_2$ (782 mg, 2.15 mmol, 0.5 equiv.) were mixed in dioxane (2 mL). Hydrazine hydrate (10.4 mL, 215 mmol, 50.0 equiv.) was added dropwise, and the reaction mixture was stirred at 60 °C for 24 h under argon. After the mixture was cooled to rt, $NaNO_2$ (5.94 g, 86.1 mmol, 20.0 equiv.) in H_2O (50mL) was added, and 1 M HCl was added slowly until the solution turned red and gas evolution had stopped (careful, toxic nitrous fumes are formed during this reaction). The mixture was extracted with CH_2Cl_2 (three times). The combined organic layers were washed with brine, dried with Na_2SO_4, and the volatiles were removed with a rotary evaporator. The crude mixture was purified by column chromatography (20% to 35% EtOAc in heptane, *v/v*) yielding tetrazine **6** as a pink solid (470 mg, 1.56 mmol, 36%). R_f = 0.15 (EtOAc/heptane, 20:80, *v/v*). ^{1}H NMR (300 MHz, $CDCl_3$) δ: 8.55 and 7.50 (AA'XX' pattern, J_{AX} = 8.1 Hz, $J_{AA'}$ = $J_{XX'}$ = 1.8 Hz, $J_{AX'}$ = 0.4 Hz, 2H), 4.97 (br s, 1H), 4.43 (d, J = 6.1 Hz, 2H), 3.09 (s, 3H), 1.48 (s, 9H). ^{13}C NMR (75 MHz, $CDCl_3$) δ: 167.4, 164.1, 156.1, 144.1, 131.0, 128.3, 128.2, 80.0, 44.6, 28.5, 21.3. LRMS (ESI+) *m/z* calcd. for $C_{10}H_{11}N_5$ $[(M-Boc)+2H)]^+$ 202.1, found: 201.9. The data agrees with the literature.[2]

(4-(6-Methyl-1,2,4,5-tetrazin-3-yl)phenyl)methanamine hydrochloride (7). Carbamate **6**

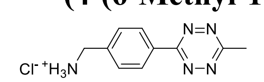

(200 mg, 0.66 mmol, 1.0 equiv.) was dissolved in CH_2Cl_2 (12 mL) and 4 M HCl in dioxane (5 mL) was added slowly. The mixture was stirred for 1 h, and the volatiles were removed under reduced pressure yielding the primary amine hydrochloride **7** as a pink solid (157 mg, 0.66 mmol, quant.) ^{1}H NMR (300 MHz, MeOD-d4) δ: 8.61 and 7.74 (AA'XX' pattern, J_{AX} = 8.1 Hz, $J_{AA'}$ = $J_{XX'}$ = 1.8 Hz, $J_{AX'}$ = 0.4 Hz, 2H), 4.28 (br s, 2H), 3.05 (s, 3H). ^{13}C NMR (75 MHz, MeOD-d4) δ: 169.0, 164.9, 138.8, 134.3, 130.9, 129.4, 44.0, 21.1. LRMS (ESI+) *m/z* calcd. for $C_{10}H_{11}N_5$ $[M+H]^+$ 202.1, found: 201.9. HRMS (ESI+) m/z calcd. for $C_{10}H_{11}N_5$ $[M+H]^+$ 202.10927, found: 202.11017. The data agrees with the literature[2]

***tert*-Butyl (1-(4-(6-methyl-1,2,4,5-tetrazin-3-yl)phenyl)-3-oxo-6,9,12,15-tetraoxa-2-azaheptadecan-17-yl)carbamate (8).** Boc-15-amino-4,7,10,13-tetraoxapentadecanoic acid (129

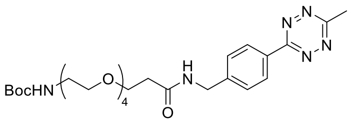

mg, 0.55 mmol, 1.0 equiv.), *N*-hydroxybenzotriazole (75 mg, 0.55 mmol, 1.0 equiv.), 1-ethyl-3-(3-dimethylaminopropyl)carbodiimide (212 mg, 1.11 mmol, 2.0 equiv.) and Et_3N (307 µl, 2.21 mmol, 4.0

equiv.) were dissolved in anhydrous DMF (12 mL) and stirred for 20 min. under argon. Tetrazine **7** (157 mg, 0.66 mmol, 1.2 equiv.) was added and the reaction mixture was stirred overnight, whereupon the solvent was removed. The mixture was purified by column chromatography (0 to 5% MeOH in CH_2Cl_2, *v/v*) yielding amide **8** as a pink oil (224 mg, 0.41 mmol, 74%). R_f =0.31 (CH_2Cl_2/MeOH, 95:5, *v/v*). ^{1}H NMR (300 MHz, $CDCl_3$) δ: 8.51 and 7.49 (AA'XX' pattern, J_{AX} = 8.1 Hz, $J_{AA'}$ = $J_{XX'}$ = 1.8 Hz, $J_{AX'}$ = 0.4 Hz, 2H), 5.02 (br s, 1H), 4.55 (d, J = 6.0 Hz, 2H), 3.77 (t, J = 5.7 Hz, 2H), 3.67 – 3.57 (m, 4H), 3.57 – 3.51 (m, 8H), 3.48 (t, J = 5.2 Hz, 2H), 3.33 – 3.20 (m, 2H), 3.07 (s, 3H), 2.56 (t, J = 5.7 Hz, 2H), 1.40 (s, 9H). ^{13}C NMR (75 MHz, $CDCl_3$) δ: 172.0, 167.3, 164.1, 156.1, 152.8, 144.0, 130.8, 128.31, 128.27, 79.4, 70.7, 70.63, 70.60, 70.5, 70.4, 70.3, 67.4, 43.1, 40.5, 37.1, 28.6, 21.3. LRMS (ESI+) *m/z* calcd. for $C_{26}H_{40}N_6O_7$ $[M+Na]^+$ 571.3, found: 571.2. HRMS (ESI+) *m/z* calcd. for $C_{26}H_{40}N_6O_7$ $[M+Na]^+$ 571.28562, found: 571.28416

1-Amino-*N*-(4-(6-methyl-1,2,4,5-tetrazin-3-yl)benzyl)-3,6,9,12-tetraoxapentadecan-15-amide hydrochloride (9). Carbamate **8** (92 mg, 0.17 mmol, 1.0 equiv.) was dissolved in CH_2Cl_2 and slowly 4 M HCl in dioxane (1.25 mL) was added. The mixture was stirred for 1.5 h, whereupon the volatiles were removed by reduced pressure yielding amine hydrochloride **9** as a pink oil (79 mg, 0.16 mmol, 97%). ^{1}H NMR (300 MHz, MeOD-d4) δ: 8.50 and 7.56 (AA'XX' pattern, J_{AX} = 8.1 Hz, $J_{AA'}$ = $J_{XX'}$ = 1.8 Hz, $J_{AX'}$ = 0.4 Hz, 2H), 4.52 (s, 2H), 3.79 (t, J = 5.9 Hz, 2H), 3.75 – 3.68 (m, 2H), 3.68 – 3.56 (m, 12H), 3.16 – 3.06 (m, 2H), 3.03 (s, 3H), 2.58 (t, J = 5.9 Hz, 2H). ^{13}C NMR (75 MHz, MeOD-d4) δ: 174.2, 168.7, 165.2, 145.1, 132.3, 129.2, 129.0, 71.4, 71.31, 71.26, 70.8, 68.3, 67.8, 43.8, 40.5, 37.5, 21.1. LRMS (ESI+) *m/z* calcd. for $C_{21}H_{32}N_6O_5$ $[M+H]^+$ 449.2, found: 449.1. HRMS (ESI+) *m/z* calcd. for $C_{21}H_{32}N_6O_5$ $[M+Na]^+$ 471.23319, found: 471.23247

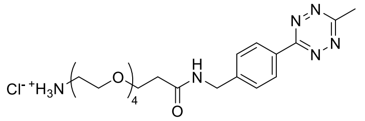

2,5-Dioxopyrrolidin-1-yl 1-(4-(6-methyl-1,2,4,5-tetrazin-3-yl)phenyl)-3,19-dioxo-6,9,12,15-tetraoxa-22,23-dithia-2,18-diazahexacosan-26-oate (3). *N,N*-Diisopropylethylamine (87 µl, 0.50 mmol, 3.1 equiv.) and tetrazine **9** (79 mg, 0.16 mmol, 1.0 equiv.) in anhydrous DMF (1 mL) were added dropwise to di(*N*-succinimidyl) 3,3'-dithiodipropionate (203 mg, 0.50 mmol, 3.1 equiv.) in anhydrous DMF (4 mL) and the reaction mixture was stirred for 1 h under argon. The solvent was removed under reduced pressure and the mixture was purified by column chromatography (0 to 10% MeOH in CH_2Cl_2, *v/v*) yielding product **3** as a pink oil (58 mg, 0.08 mmol, 48%). R_f = 0.55 (CH_2Cl_2/MeOH, 9:1, *v/v*). ^{1}H NMR (500 MHz, $CDCl_3$) δ: 8.52 and 7.51 (AA'XX' pattern, J_{AX} = 8.1 Hz, $J_{AA'}$ = $J_{XX'}$ = 1.8 Hz, $J_{AX'}$ = 0.4 Hz, 2H), 7.17 (br s, 1H), 6.61 (br s, 1H), 4.56 (d, J = 5.9 Hz, 2H), 3.79 (t, J = 5.6 Hz, 2H), 3.69 – 3.59 (m, 4H), 3.59 – 3.53 (m, 8H), 3.51 (t, J = 5.0 Hz, 2H), 3.43 – 3.38 (m, 2H), 3.08 (s, 3H), 3.06 – 3.01 (m, 2H), 3.00 – 2.92 (m, 4H), 2.85 – 2.81 (m, 4H), 2.60 – 2.53 (m, 4H). ^{13}C NMR (126 MHz, $CDCl_3$) δ: 171.9, 170.9, 169.1, 167.3, 167.2, 164.0, 143.9, 130.8, 128.3, 128.2, 70.6, 70.55, 70.53, 70.4, 70.3, 70.2, 70.0, 67.5, 43.1, 39.4, 37.1, 35.9, 33.9, 32.2, 31.2, 25.7, 21.3. LRMS (ESI+) *m/z* calcd. for $C_{31}H_{43}N_7O_{10}S_2$ $[M+Na]^+$ 760.2, found: 760.1. HRMS (ESI+) *m/z* calcd. for $C_{31}H_{43}N_7O_{10}S_2$ $[M+Na]^+$ 760.24105, found: 760.23943.

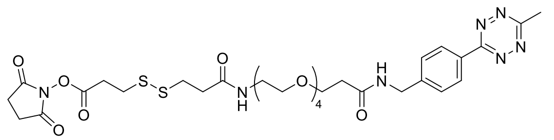

3. NMR spectra

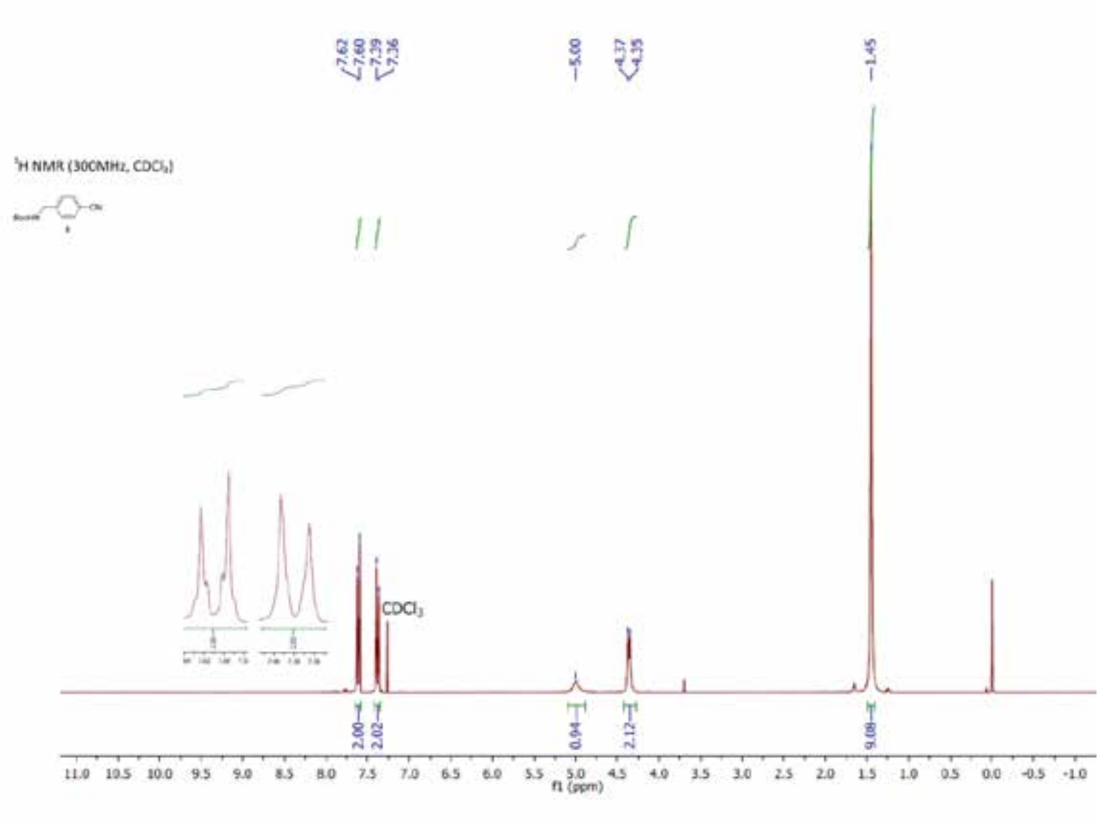

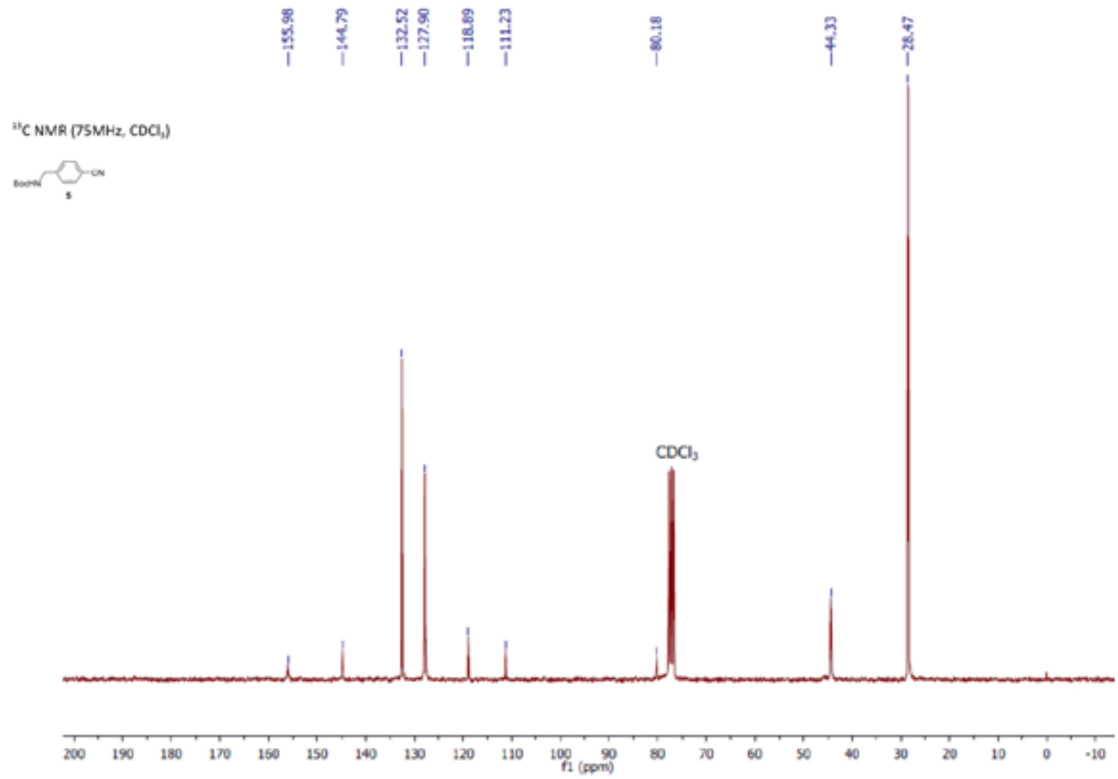

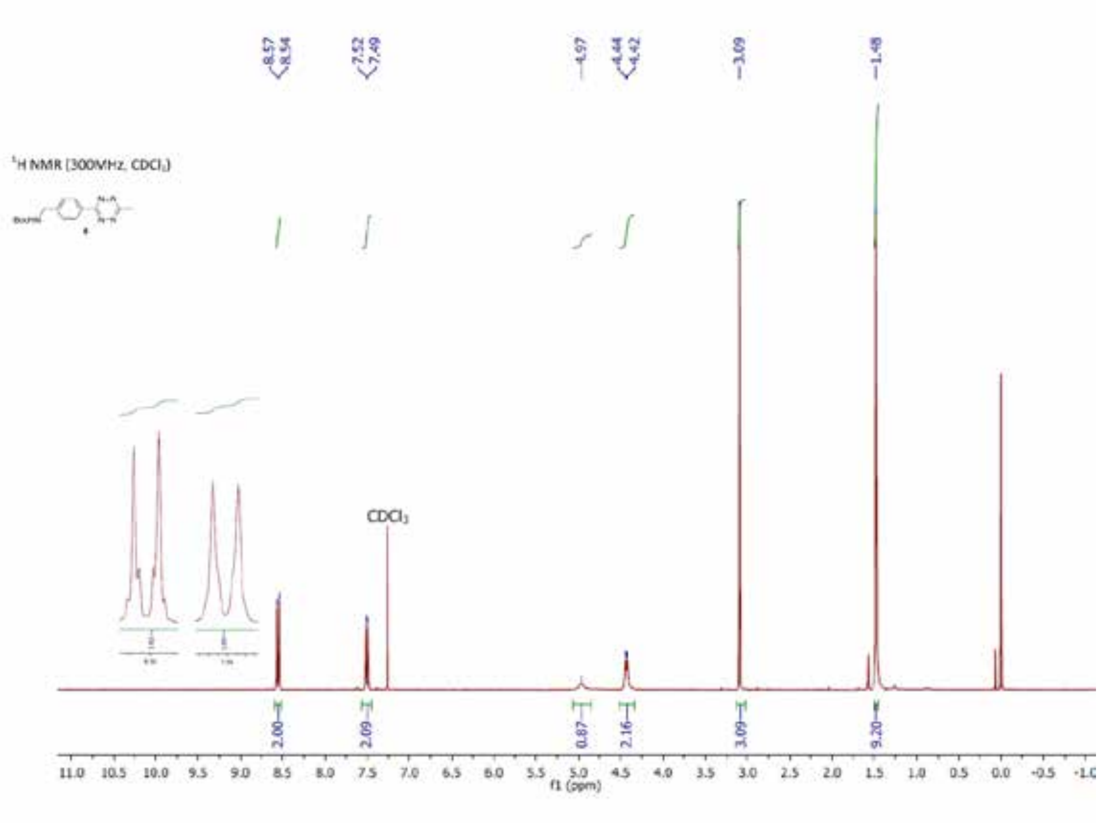

8.57
8.54
7.52
7.49
4.97
4.44
4.42
3.09
1.48
^{1}H NMR (300MHz, CDCl$_3$)
CDCl$_3$
2.00
2.09
0.87
2.16
3.09
9.20
f1 (ppm)

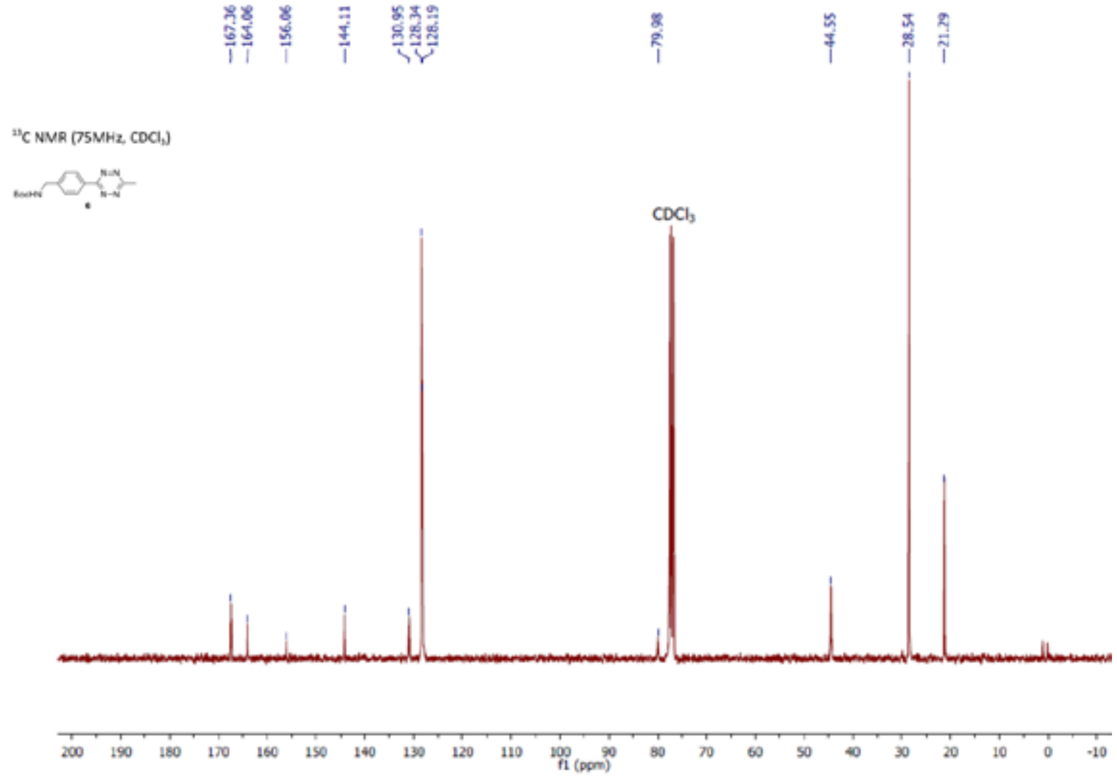

167.36
164.06
156.06
144.11
130.95
128.34
128.19
79.98
44.55
28.54
21.29
^{13}C NMR (75MHz, CDCl$_3$)
CDCl$_3$
f1 (ppm)

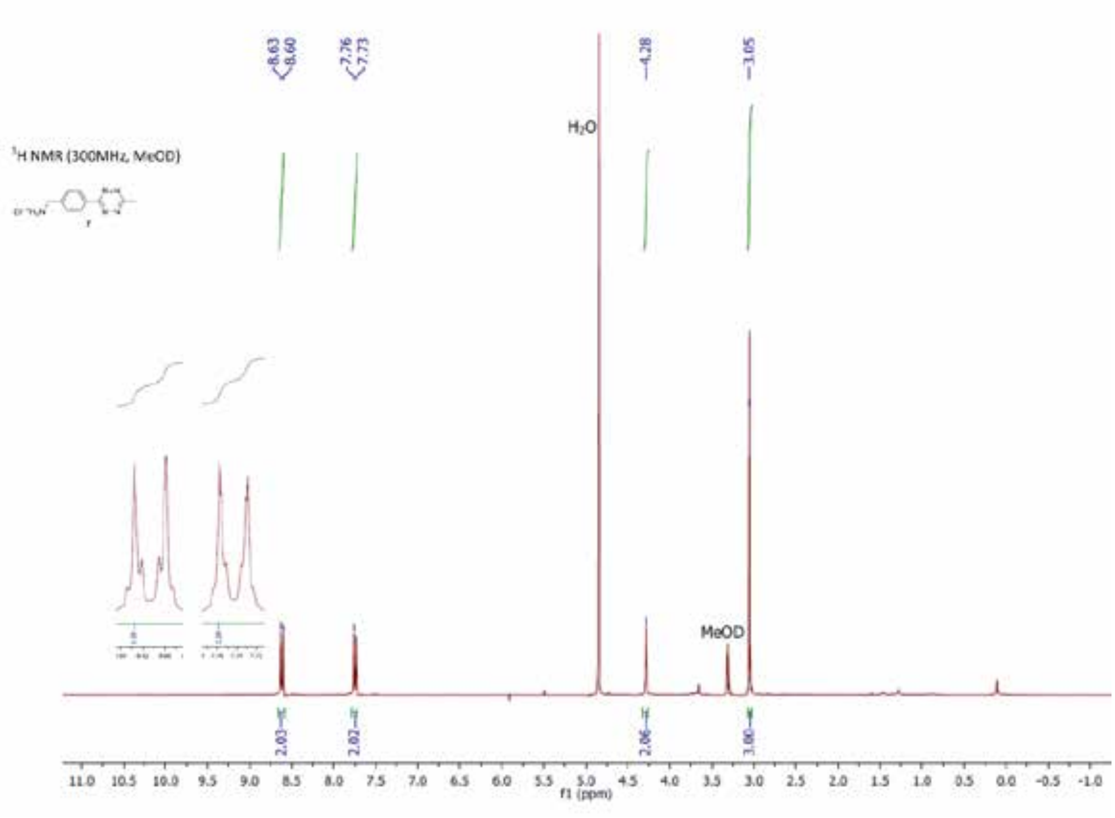
1H NMR (300MHz, MeOD)
8.63
8.60
7.76
7.73
4.28
3.05
H2O
MeOD
2.03
2.02
2.06
3.00
f1 (ppm)

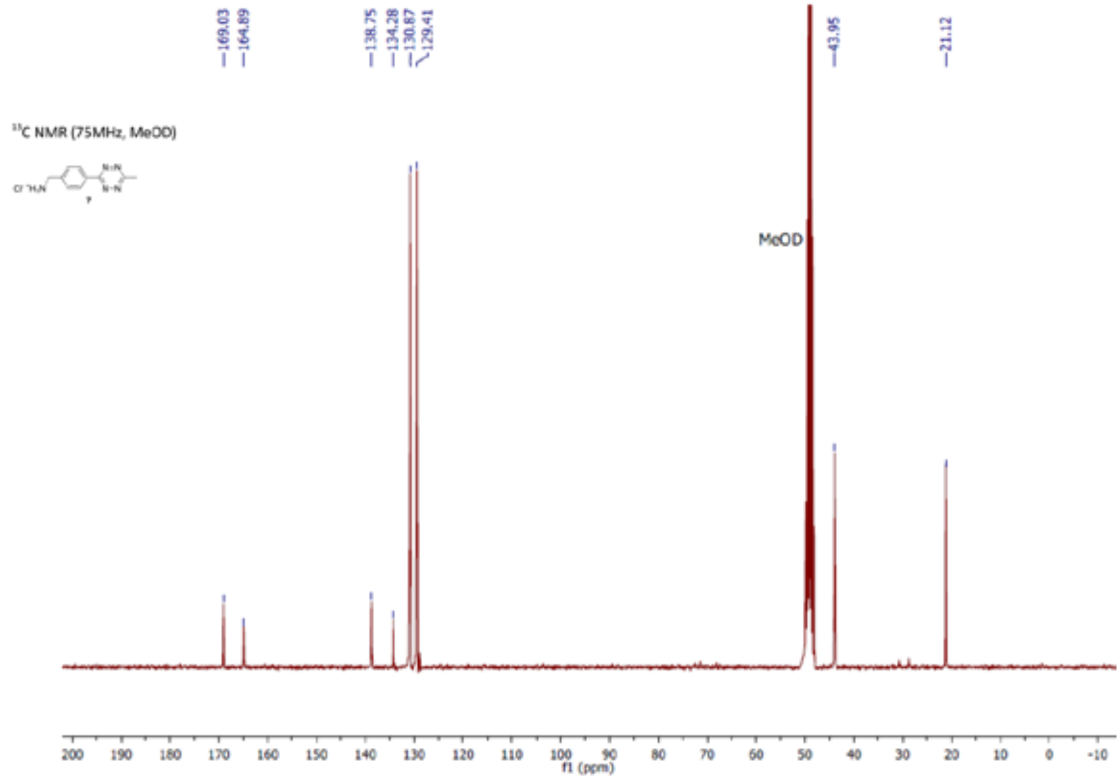
13C NMR (75MHz, MeOD)
169.03
164.89
138.75
134.28
130.87
129.41
43.95
21.12
MeOD
f1 (ppm)

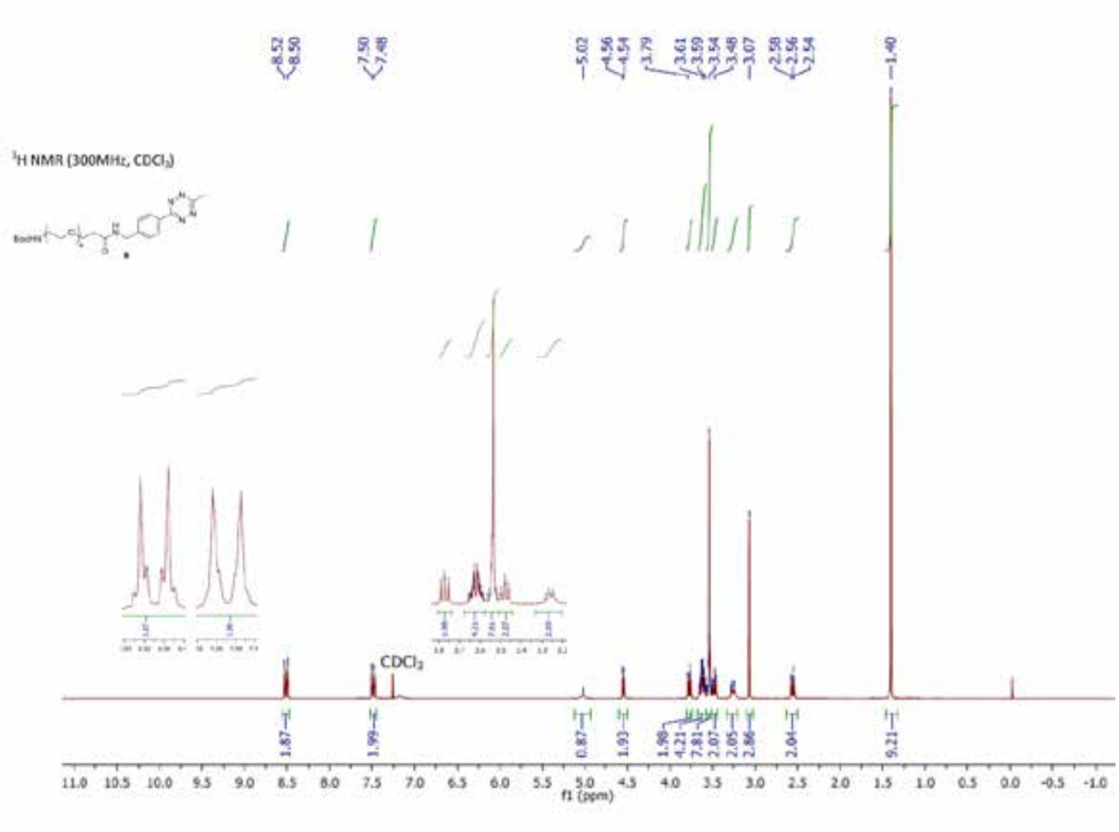

^{1}H NMR (300MHz, CDCl$_3$)
CDCl$_3$
f1 (ppm)

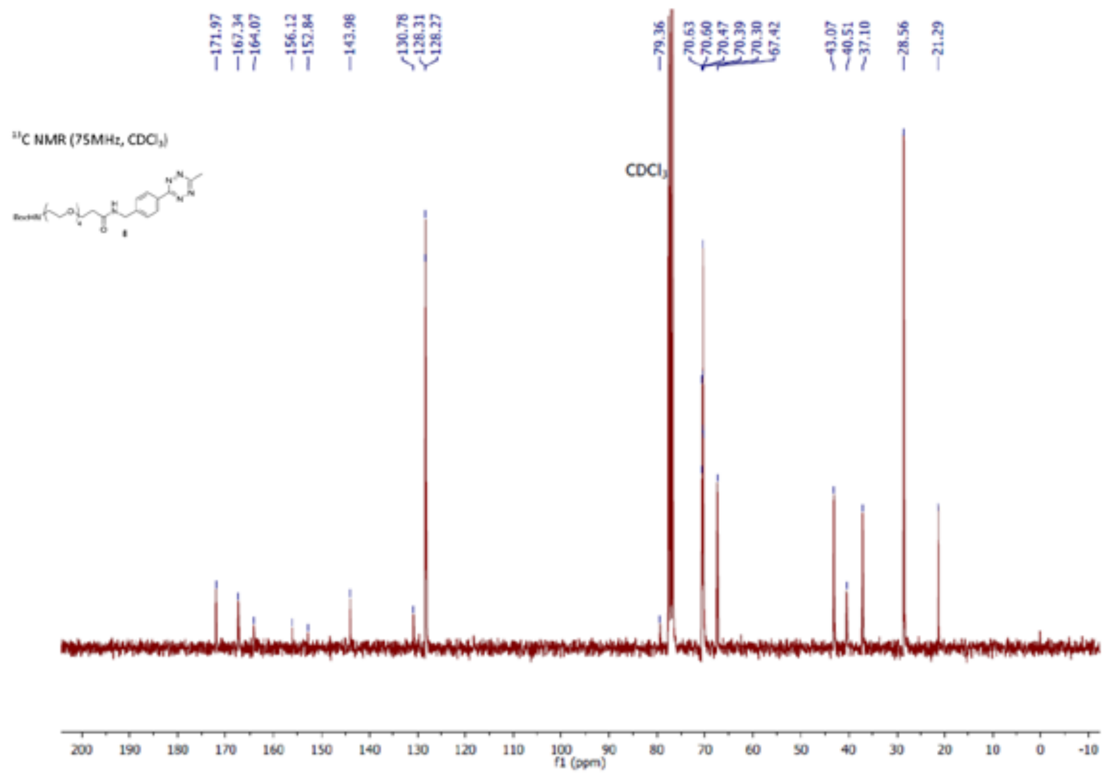

^{13}C NMR (75MHz, CDCl$_3$)
CDCl$_3$
f1 (ppm)

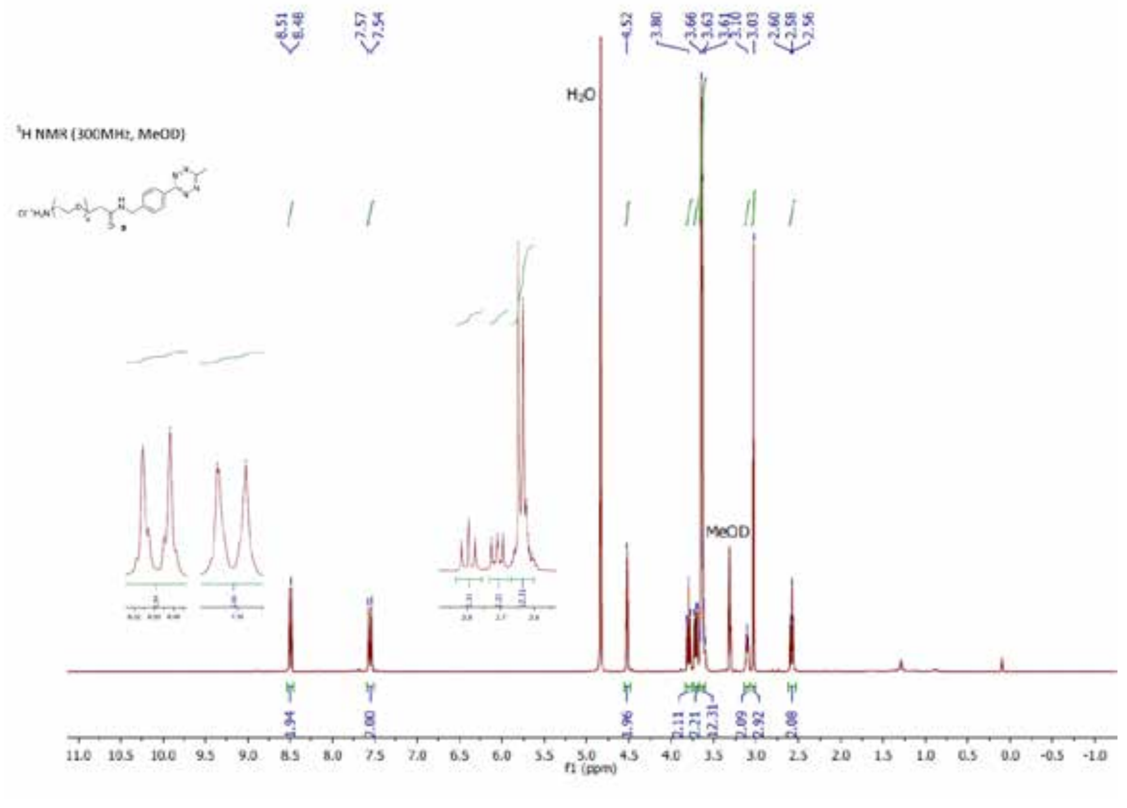

^{1}H NMR (300MHz, MeOD)
H2O
MeOD
8.51
8.48
7.57
7.54
4.52
3.80
3.66
3.63
3.61
3.10
3.03
2.60
2.58
2.56
1.94
2.00
1.96
2.11
2.21
12.31
2.09
2.92
2.08
f1 (ppm)

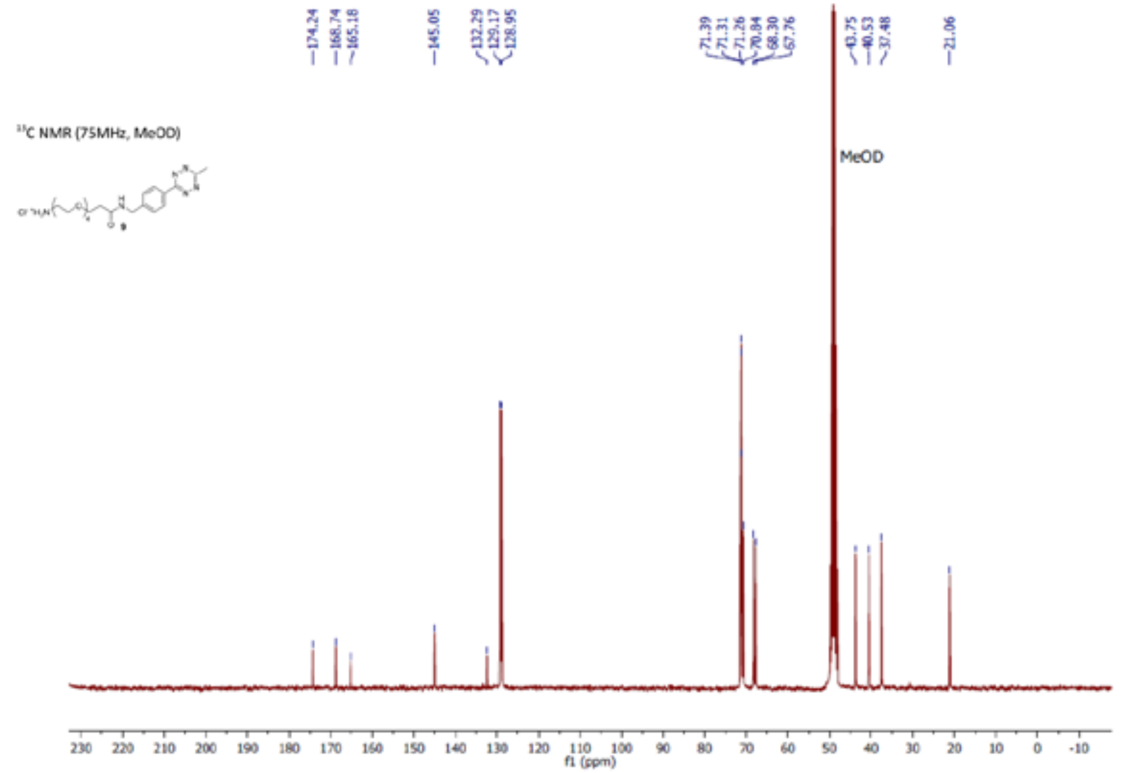

^{13}C NMR (75MHz, MeOD)
MeOD
174.24
168.74
165.18
145.05
132.29
129.17
128.95
71.39
71.31
71.26
70.84
68.30
67.76
43.75
40.53
37.48
21.06
f1 (ppm)

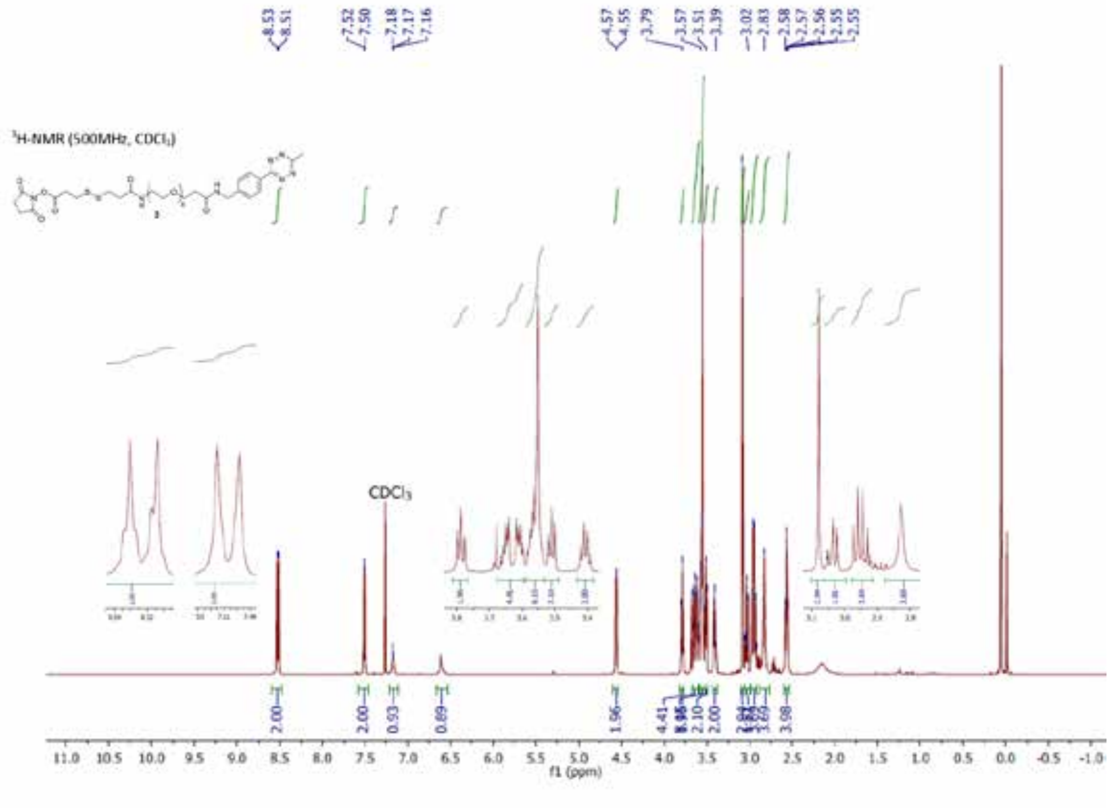
1H-NMR (500MHz, CDCl3)
CDCl3
f1 (ppm)

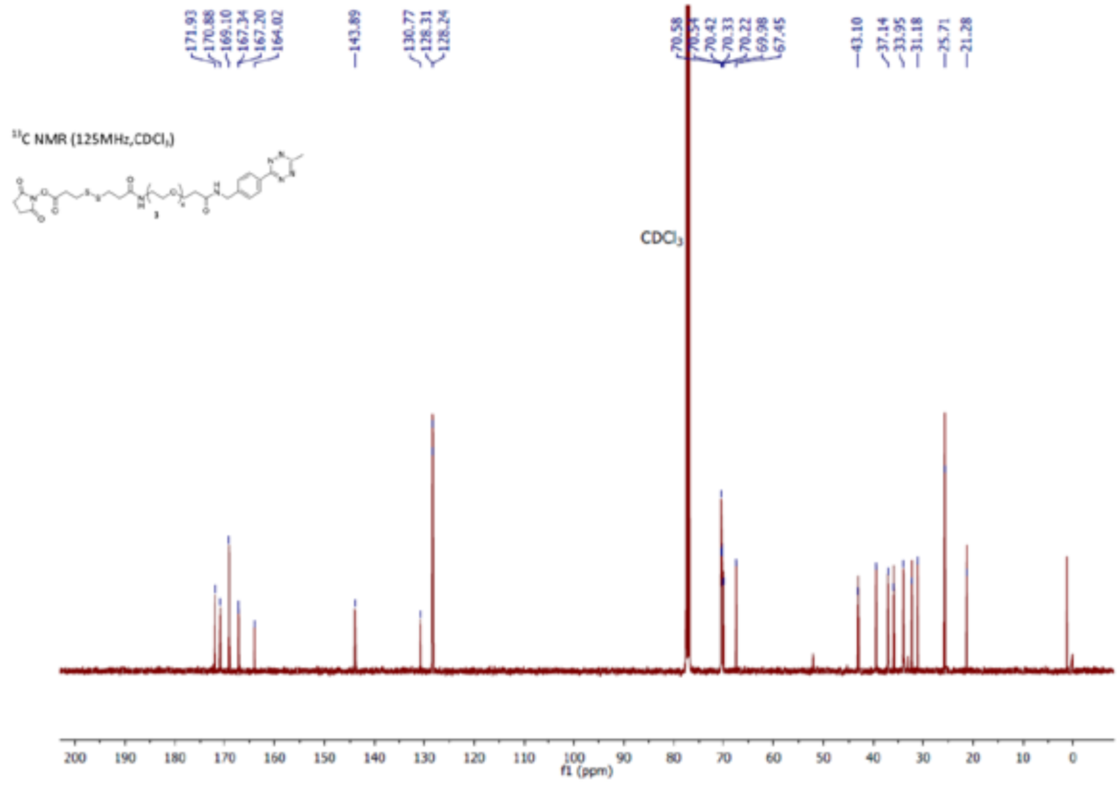
13C NMR (125MHz,CDCl3)
CDCl3
f1 (ppm)

Supporting Figures

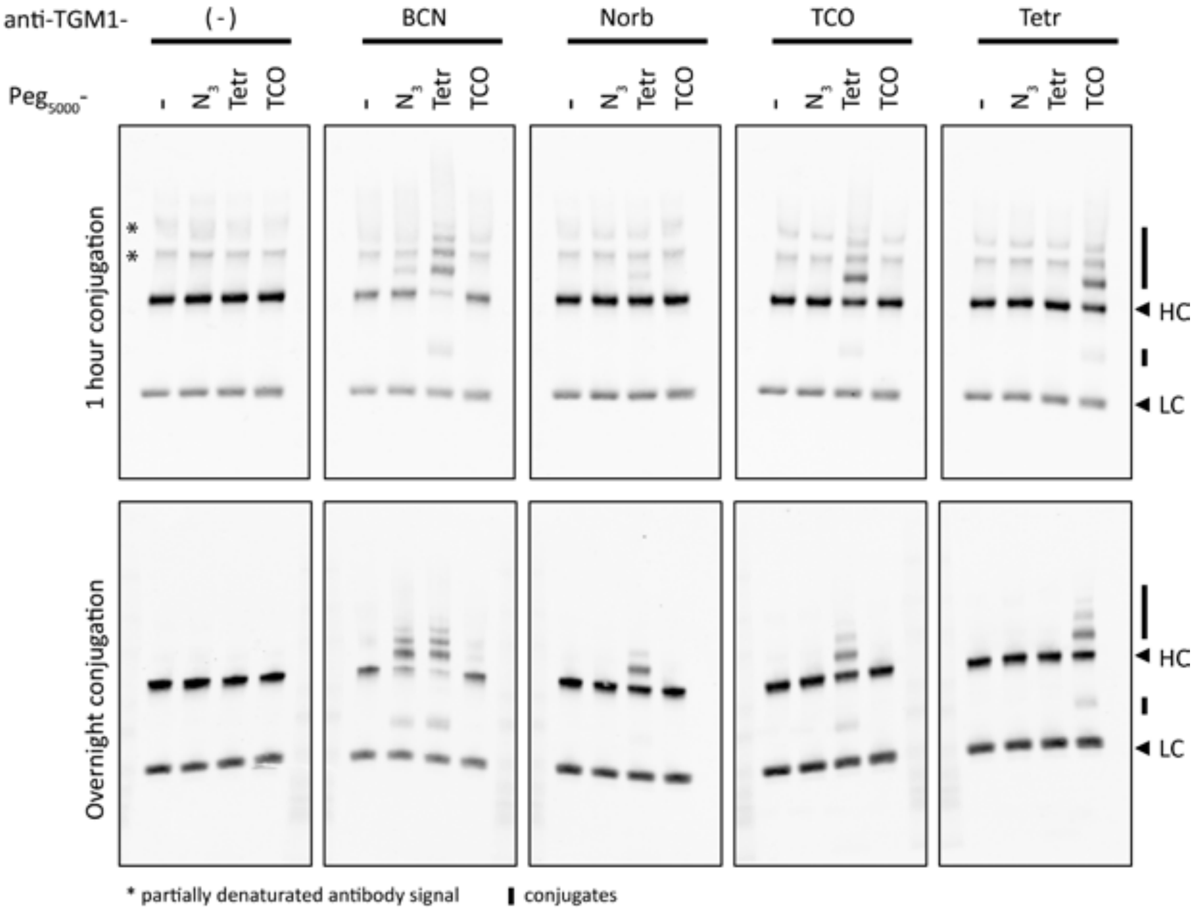

Figure S1. Comparing different conjugation strategies for the conjugation of a mouse antibody (BC.1) with PEG_{5000}. Western-blot analysis after 1 hour or overnight conjugation reactions. Abbreviations: BCN: Bicyclononyne, Norb: Norbornene, TCO: *trans*-Cyclooctene, Tetr: Tetrazine, HC: Heavy Chain, LC: Light Chain. (* indicates partially denaturated antibody signal. | indicates conjugated antibody)

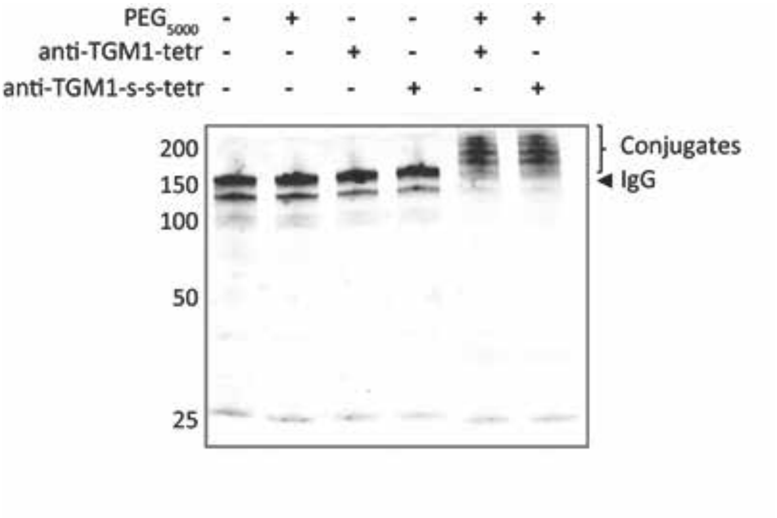

Figure S2. Western-blot analysis of non-cleavable linker **1** and cleavable linker **3** functionalized mouse antibody anti-TGM1 after overnight conjugation with TCO-PEG_{5000}.

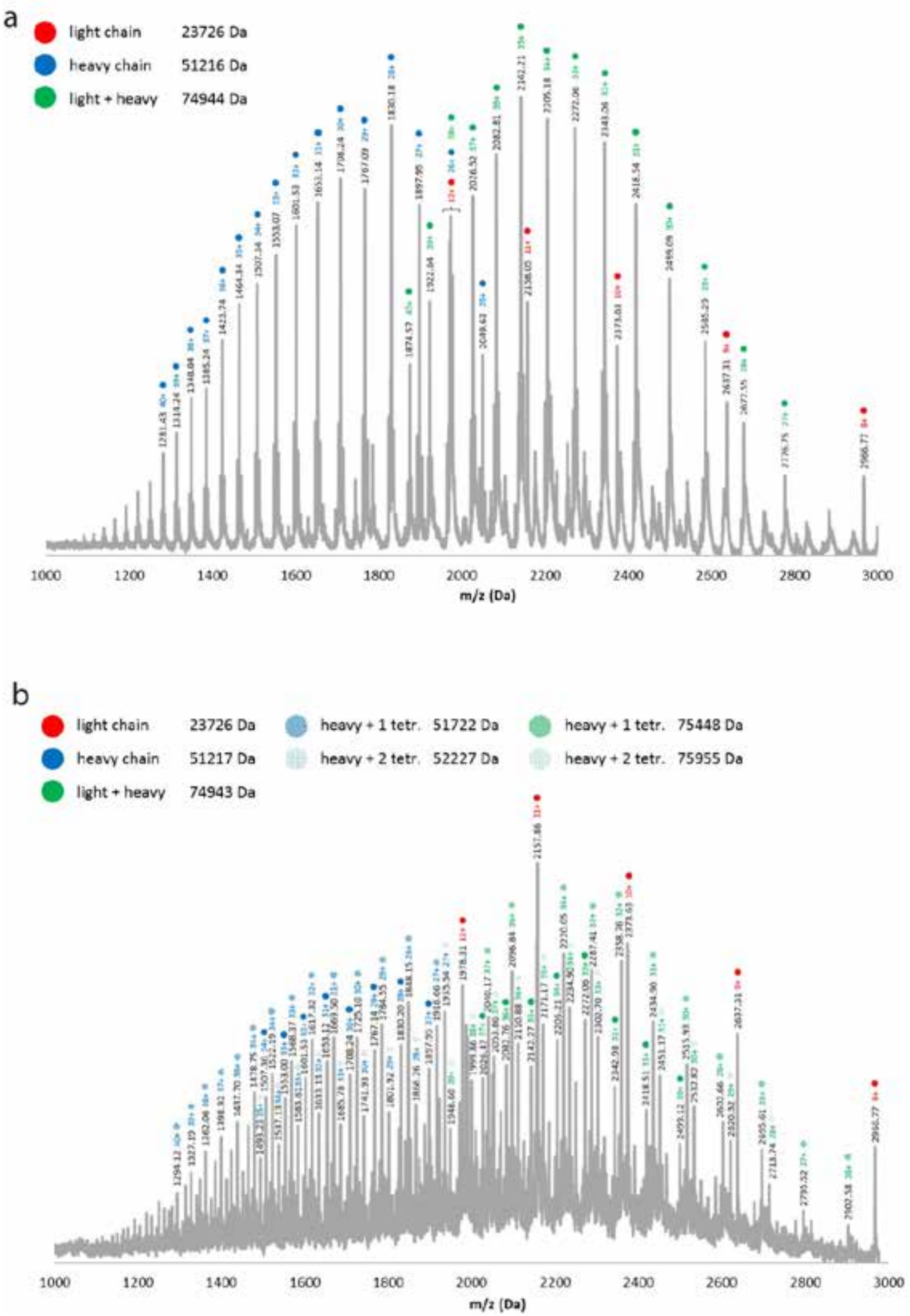

Figure S3. Multiply charged ion series of unfunctionalized (a) and tetrazine-functionalized (b) anti-TGM1 antibody, measured by ESI-TOF mass spectrometry.

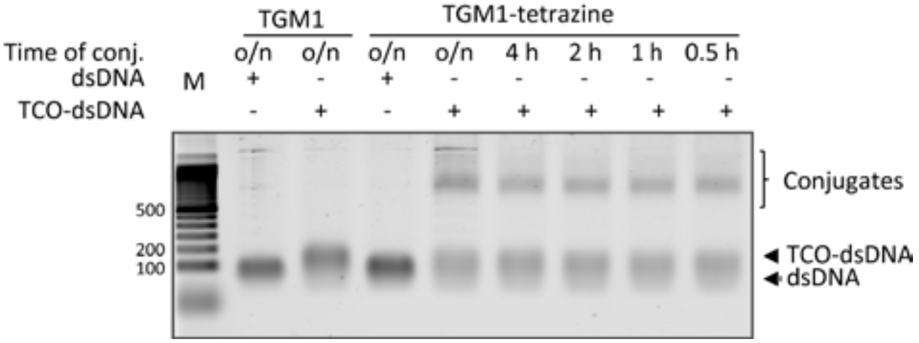

Figure S4. Agarose gel and ethidium bromide stain of dsDNA and anti-TGM1 antibody conjugates of time series of conjugation.

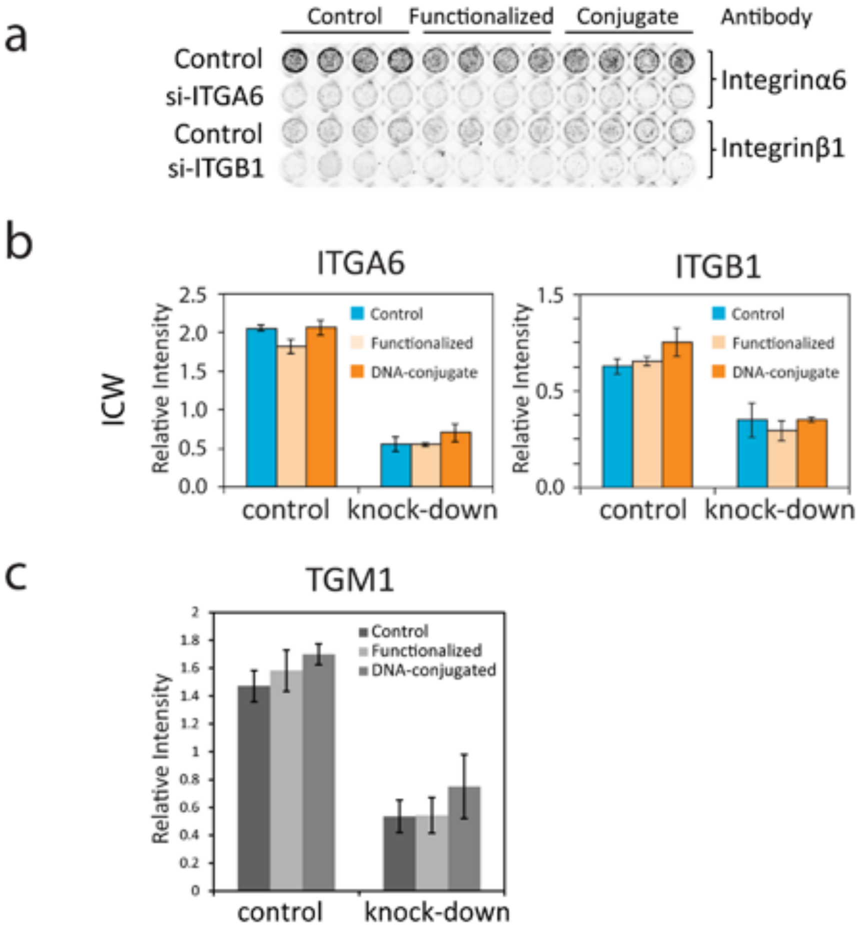

Figure S5. Comparing specificity in immuno-staining experiments of tetrazine-functionalized and DNA-conjugated antibodies against integrin alpha-6 (ITGA6) and integrin beta-1 (ITGB1). (a) Fluorescent secondary antibody signal after immuno-staining of siRNA control or siRNA ITGA6 or siRNA ITGB1 transfected keratinocyte cell populations. (b) Quantification of signal normalized over DRAQ5 signal to correct for cell number variability. (c) Normalized TGM1 signal over DRAQ5 (N=3). Experiment was performed on keratinocyte cell populations that were treated with serum for 48 hours to induce differentiation.

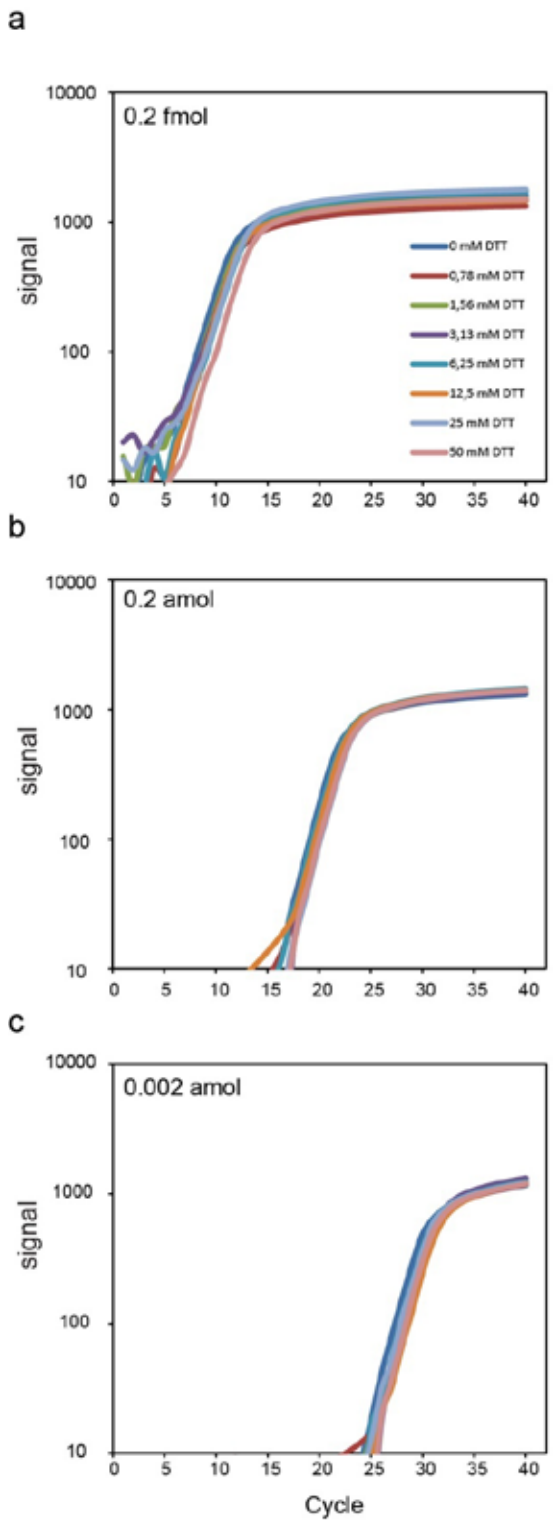

Figure S6. Signal in qPCR of reactions with specific concentration of DTT (mM) and specific amount of DNA template: a) 0.2 fmol, b) 0.2 amol, c) 0.002 amol.

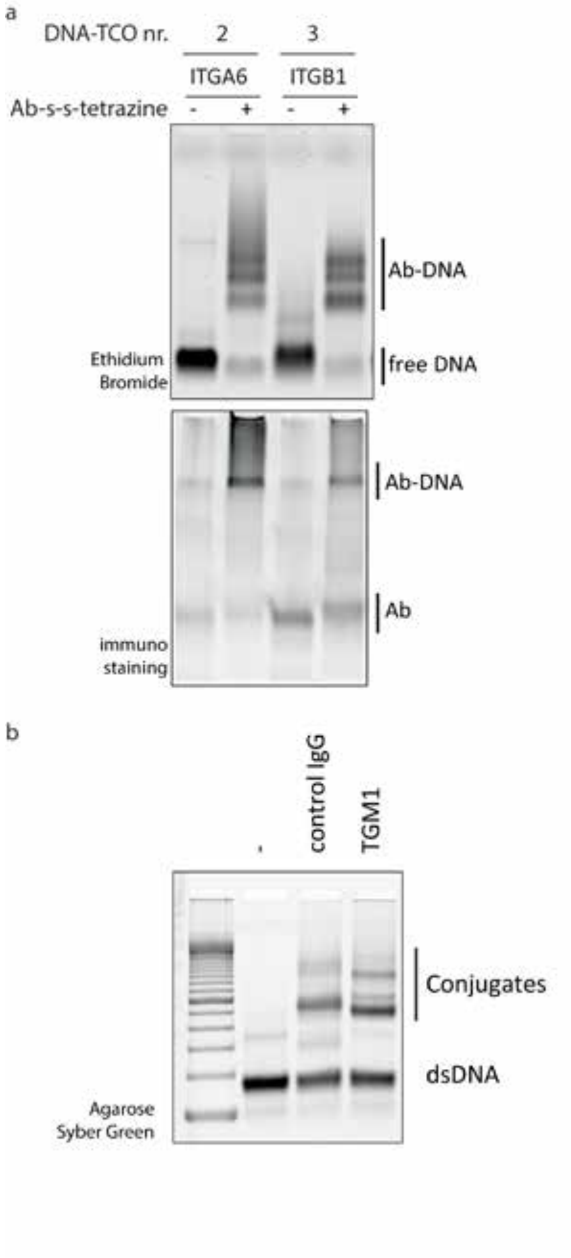

Figure S7. Visualization of conjugates. (a) Immunostaining (in gel western) and ethidium bromide staining of antibodies (ITGA6 and ITGB1) and dsDNA (barcode-2 and 3), respectively, in polyacrylamide gel. ITGA6 or ITGB1 functionalized or non-functionalized antibodies were used in the conjugation reactions. (b) Agarose gel with control dsDNA (Barcode-1) and dsDNA (Barcode-1) conjugated with control IgG (Rabbit) and BC.1 (against TGM1), stained with Syber Green.

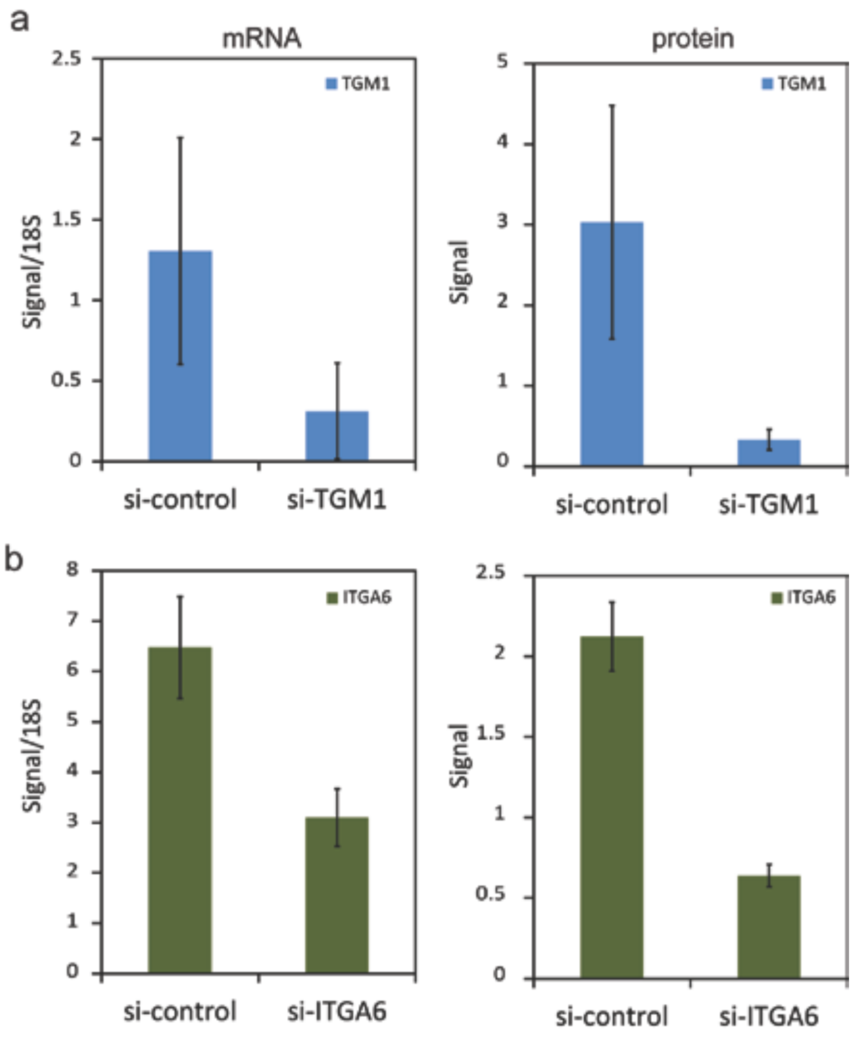

Figure S8. qPCR signal of mRNA levels via RT-qPCR and protein levels via immuno-PCR. a) TGM1 mRNA (left, $2^{-Ct}\times10^{-4}$, p = 0.0016) and protein levels (right, $2^{-Ct}\times10^{-3}$, p= 0.033). b) ITGA6 mRNA (left, $2^{-Ct}\times10^{-5}$ p = 0.0073) and protein levels (right, $2^{-Ct}\times10^{-7}$, p = 0.0003)

Name	5' -> 3' sequence	Source
Barcode-1	AATGATACGGCGACCACCGAGATCTACACTCTTTCCCTACACGACGCTCTTCCGATCTNNNNNNNCGACGAATCAGTCAACAGATAAGCGAGCAAGATCGGAAGAGCACACGTCTGAACTCCAGTCAC	Biolegio
Barcode_1 FW primer	AATGATACGGCGACCACCGA	Biolegio
Barcode_1 RV primer	GTGACTGGAGTTCAGACGTG	Biolegio
Barcode-2	TGGCTGCTGTGAATGCTTACcttgtgtaagctttaaatggtatcagtactgagattaactgccccccttcatggttctcacctcagattcaagctgtaatccccctgtattgtttacacCTGTTAGGCCTGTCCTGCTC	*Xenopus Tropicalis* isolated genomic DNA
Barcode_2 FW primer	TGGCTGCTGTGAATGCTTAC	Biolegio
Barcode_2 RV primer	GAGCAGGACAGGCCTAACAG	Biolegio
Barcode-3	GCATAGGGCAGGCAGTACATACAGTGACACAATGCTGGCACTGCTCCTACAGCCTGGTTGTAAACAGCTGTATAATGTGGGTGCTTACAGTCTGATCCTGAGTTGTGAACAATGCAGGGACAAACAGGTGT	*Xenopus Tropicalis* isolated genomic DNA
Barcode_3 FW primer	GCATAGGGCAGGCAGTACAT	Biolegio
Barcode_3 RV primer	ACACCTGTTTGTCCCTGCAT	Biolegio

Supplemental table 1. Overview of oligo sequences for the production of barcode 1, 2 or 3 for GAPDH, ITGA6 or ITGB1 conjugates, respectively. N: A, T, G or C random sequence (unique molecular identifier)

References

(1) Lang, K., Davis, L., Wallace, S., Mahesh, M., Cox, D. J., Blackman, M. L., Fox, J. M., and Chin, J. W. (2012) Genetic encoding of bicyclononynes and trans-cyclooctenes for site-specific protein labeling in vitro and in live mammalian cells via rapid fluorogenic Diels-Alder reactions. *J. Am. Chem. Soc. 134*, 10317–10320.

(2) Evans, H. L., Nguyen, Q.-D., Carroll, L. S., Kaliszczak, M., Twyman, F. J., Spivey, A. C., and Aboagye, E. O. (2014) A bioorthogonal ^{68}Ga-labelling strategy for rapid in vivo imaging. *Chem. Commun. (Camb).* 9557–9560.
